# Supplementary material for: PseudotimeDE: inference of differential gene expression along cell pseudotime with well-calibrated p-values from single-cell RNA sequencing data
Source: Genome Biol. 2021 Apr 29;22:124. doi: 10.1186/s13059-021-02341-y (PMC8082818; doi:10.1186/s13059-021-02341-y)
Supplement: Supplementary file 1 — Additional file 1 Supplementary materials. It includes all supplementary text and figures. [file 13059_2021_2341_MOESM1_ESM.pdf]

Supplementary Materials of "PseudotimeDE: inference of differential gene expression along cell pseudotime with well-calibrated p-values from single-cell RNA sequencing data"

Dongyuan Song and Jingyi Jessica Li

Goodness-of-fit of the parametric distribution

We perform a goodness-of-fit test to show that our parametric approach fits the empirical distributions well (Fig. S21). We choose the Anderson-Darling (AD) test since it gives more weights on the tail of the distribution than the Kolmogorov-Smirnov test does. On one simulated dataset and one real dataset, the distribution of AD test p-values is approximately Uniform$[0, 1]$ (Fig. S21a & c), indicating that the parametric distributions fit the empirical null distributions well (i.e., all cases are from the null). Since this goodness-of-fit check is crucial, `PseudotimeDE` R package automatically outputs the AD test p-value per gene so that users can check whether the parametric approximation works well; if not, users may increase the number of subsampling and use the empirical null for DE p-value calculation.

Choice of the subsampling proportion

In general, results of PseudotimeDE is robust to the subsampling proportion. We have applied PseudotimeDE to one simulation dataset (Fig. S22) with five sampling proportions (50%, 60%, 70%, 80%, and 90%), among which 80% is the default used in PseudotimeDE. From the results, we first observe that the p-values (on the $-\log_{10}$ scale) are highly correlated between 80% and any other proportion (Fig. S22c), confirming the robustness of PseudotimeDE to the subsampling proportion. Second, we observe that the power of PseudotimeDE remains stable under varying sampling proportions, though the power is slightly lower when the sampling proportion is 50% (Fig. S22a). Third, the false discovery proportion (FDP) of PseudotimeDE is also stable under varying sampling proportions, though the FDP is slightly higher than the nominal level when the sampling proportion is 90% (Fig. S22b). Therefore, we conclude that 80% is a reasonable default sampling proportion.

Differences of the NB-GAM fitting in PseudotimeDE, tradeSeq, and NBAMSeq

PseudotimeDE, tradeSeq, and NBAMSeq all rely on the R package `mgcv` for fitting NB-GAM by penalized-restricted maximum likelihood. Therefore, the model fitting of NB-GAM is overall similar in the three methods. There are only minor differences, listed below.

1. In PseudotimeDE, the dispersion parameter ϕ_j of gene j is estimated simultaneously with the mean parameter μ_{ij} of gene j in cell i by the penalized-restricted maximum likelihood (PRML); in contrast, NBAMSeq uses the maximum a posterior (MAP) dispersion estimate calculated by R package `DESeq2` [1]. NBAMSeq is designed for bulk RNA-seq and accounts for a samll sample size (i.e., number of replicates); therefore, the MAP estimate by `DESeq2` is more biased (for variance reduction purpose) than the PRML estimate used by PseudotimeDE. Given that scRNA-seq has a large enough sample size (i.e., number of cells), we think that the PRML estimate of ϕ_j is preferred over the MAP estimate.

2. In PseudotimeDE, the number of knots K is pre-defined as a fix number (the default $K = 6$, which is usually large enough). In contrast, tradeSeq randomly subsamples a small set of genes to choose a K for all genes.

3. PseudotimeDE fits a NB-GAM to each lineage, while tradeSeq incorporates a lineage covariate so that it can fit a NB-GAM to multiple lineages jointly. The reason of this difference is that PseudotimeDE needs to account for the uncertainty in pseudotime inference, and within-lineage model fitting makes the inference easier.

4. Pseudotime also implements a zero-inflated NB-GAM (ZINB-GAM) that includes a dropout probability parameter for each gene, and PseudotimeDE estimates this parameter internally. In contrast, while tradeSeq can also turn its NB-GAM into ZINB-GAM, it estimates the dropout probability parameters externally by ZINB-WaVE [2]; NBAMSeq is designed for bulk RNA-seq data and does not consider zero inflation.

Although the NB-GAM fitting of PseudotimeDE is similar to that of the other two methods, its inference (p-value calculation) is completely different, and this is the main novelty of PseudotimeDE.

The number of knots K

The number of knots, K, a parameter in the generalized additive model (GAM), defines the number and positions of piecewise polynomial functions in a spline function.

$$f_j(T_i) = \sum_{k=1}^{K} b_k(T_i)\beta_{jk}$$

where $f_j(\cdot)$ is the smooth spline function of gene j, T_i is the pseudotime of cell i, and K is the number of knots. It is worth noting that $K-1$ is also the **nominal degree of freedom** of the model.

K needs to be specified before the negative binomial-GAM (NB-GAM) is fitted. For the GAM, "exact choice of K is not generally critical [3]," if the GAM is correctly used for inference. This is why we do not include the K selection step in PseudotimeDE. The default $K = 6$ in PseudotimeDE is usually large enough for modelling gene trajectories. Using a larger K is not harmful, but it will increase the computational time.

In contrast, tradeSeq has a K selection step, which we think is problematic. To choose K, tradeSeq randomly samples a small set of genes and uses AIC to find an "appropriate" K for all genes (see *Choosing an appropriate number of knots* in the tradeSeq paper [4]). We follow their vignette and obtain $K = 4$ in Fig. 3a and $K = 6$ in Fig. 3f. In tradeSeq, the null distribution of the test statistic S_j is

$$S_j \sim \chi^2_{K-1}$$

Therefore, when $K = 4$ (Fig. 3a), the null distribution of every gene is χ^2_3; when $K = 6$ (Fig. 3f), the null distribution of every gene is χ^2_5. As a result, the distributions of tradeSeq p-values under the null are quite different between Fig. 3a and Fig. 3f. It seems that the result of tradeSeq is highly sensitive to the choice of K. Such sensitivity is probably due to the incorrect null distribution. That is, tradeSeq uses the nominal degree of freedom $K - 1$ in the null distribution (χ^2_{K-1}); however, the correct way is to use the effective degree of freedom, which is gene-specific and always smaller than $K-1$ due to the penalization on the likelihood function. As a result, tradeSeq uses null distributions (of its test statistics) that have heavier right tails than those of the correct null distributions, resulting in conservative p-values (whose distribution under the null has a mode near 1). Details about the theory and usage of the effective degree of freedom in GAM can be found in Dr. Simon Woods' book [5] and 2013 *Biometrika* paper [6].

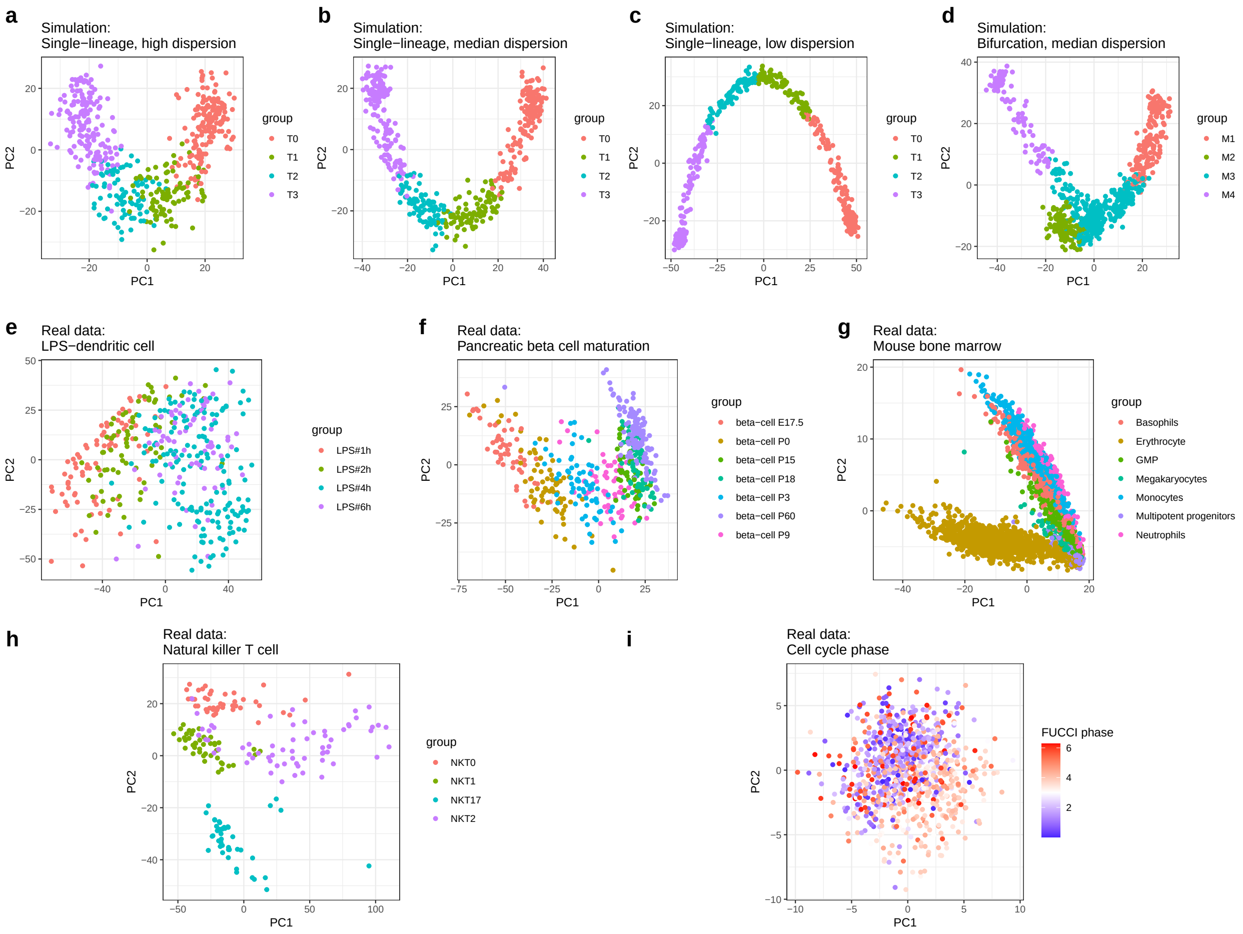

Figure S1: PCA visualization of datasets. PCA visualization of all synthetic and real datasets used in this paper. Panels (a)–(d) are synthetic datasets, where the groups correspond to time points. Panels (e)–(h) are real datasets, where the groups correspond to time points in (e) & (f) or annotated cell types in (g) & (h). Panel (i) is a real dataset with external cell cycle information, where the FUCCI phase indicates experimentally measured cell cycle phase.

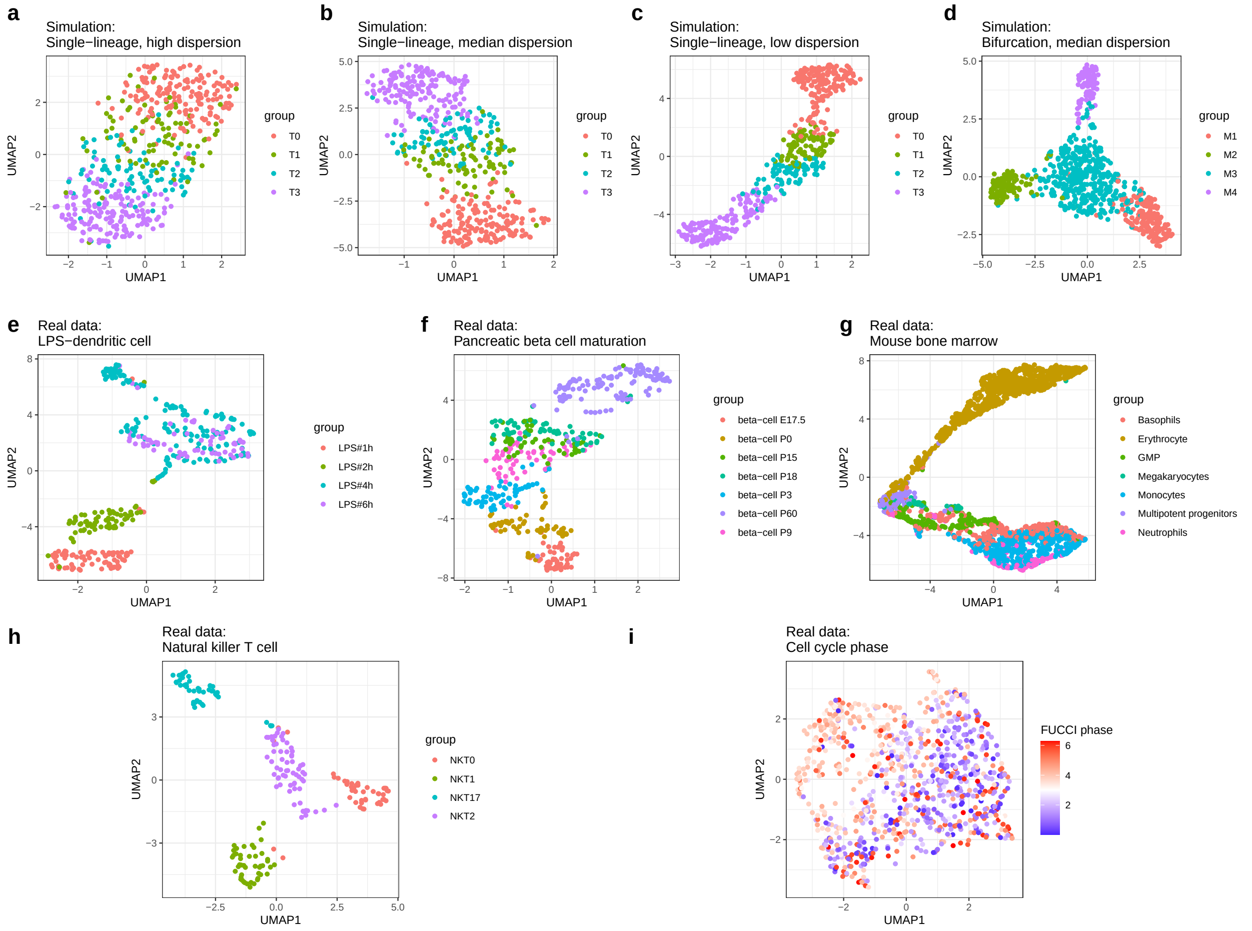

Figure S2: UMAP visualization of datasets. UMAP visualization of all synthetic and real datasets used in this paper. Panels (a)–(d) are synthetic datasets, where the groups correspond to time points. Panels (e)–(h) are real datasets, where the groups correspond to time points in (e) & (f) or annotated cell types in (g) & (h). Panel (i) is a real dataset with external cell cycle information, where the FUCCI phase indicates experimentally measured cell cycle phase.

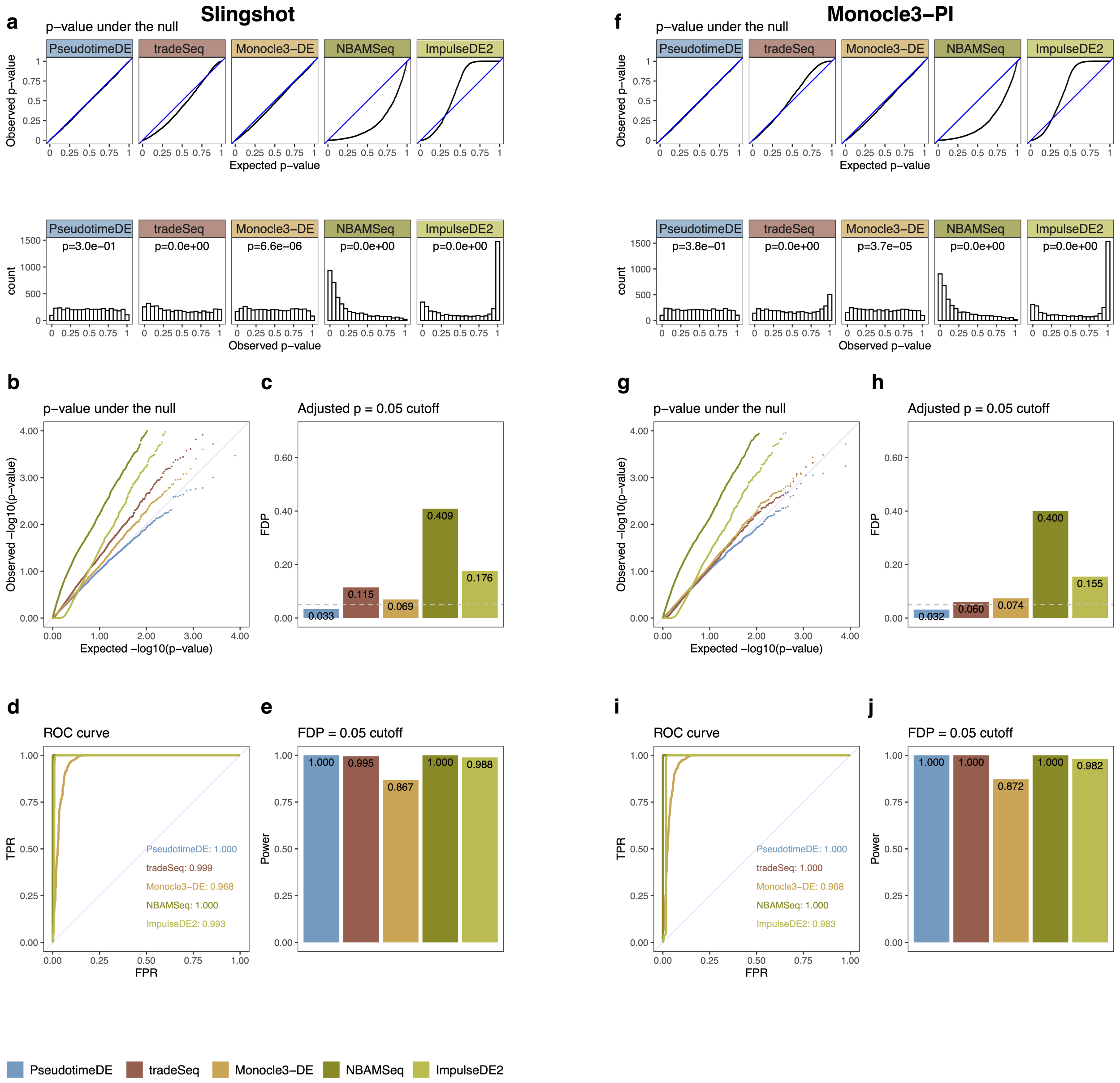

Figure S3: Comparison of five methods (PseudotimeDE, tradeSeq, Monocle3-DE, NBAMSeq, ImpulseDE2) for identifying DE genes along cell pseudotime on synthetic single-lineage data with low dispersion. Left panels (a)–(e) are based on pseudotime inferred by Slingshot; right panels (f)–(j) are based on pseudotime inferred by Monocle3-PI. **(a) & (f)** Distributions of non-DE genes' observed p-values by five DE methods with inferred pseudotime. Top: quantile-quantile plots that compare the empirical quantiles of the observed p-values against the expected quantiles of the Uniform$[0,1]$ distribution. Bottom: histograms of the observed p-values. The p-values shown on top of histograms are from the Kolmogorov–Smirnov test under the null hypothesis that the distribution is Uniform$[0,1]$. The larger the p-value, the more uniform the distribution is. Among the five DE methods, PseudotimeDE's observed p-values follow most closely the expected Uniform$[0,1]$ distribution. **(b) & (g)** Quantile-quantile plots of the same p-values as in (a) and (f) on the negative $\log_{10}$ scale. PseudotimeDE returns better-calibrated small p-values than the other four methods do. **(c) & (h)** FDPs of the five DE methods with the target FDR 0.05 (BH adjusted-$p \leq 0.05$). PseudotimeDE yields the FDP below 0.05, while other methods do not. **(d) & (i)** ROC curves and AUROC values of the five DE methods. Since the dispersion of data is extremely low, all methods achieves high AUROC. **(e) & (j)** Power of the five DE methods under the FDP = 0.05 cutoff. Due to the same low dispersion reason, all methods achieves high power.

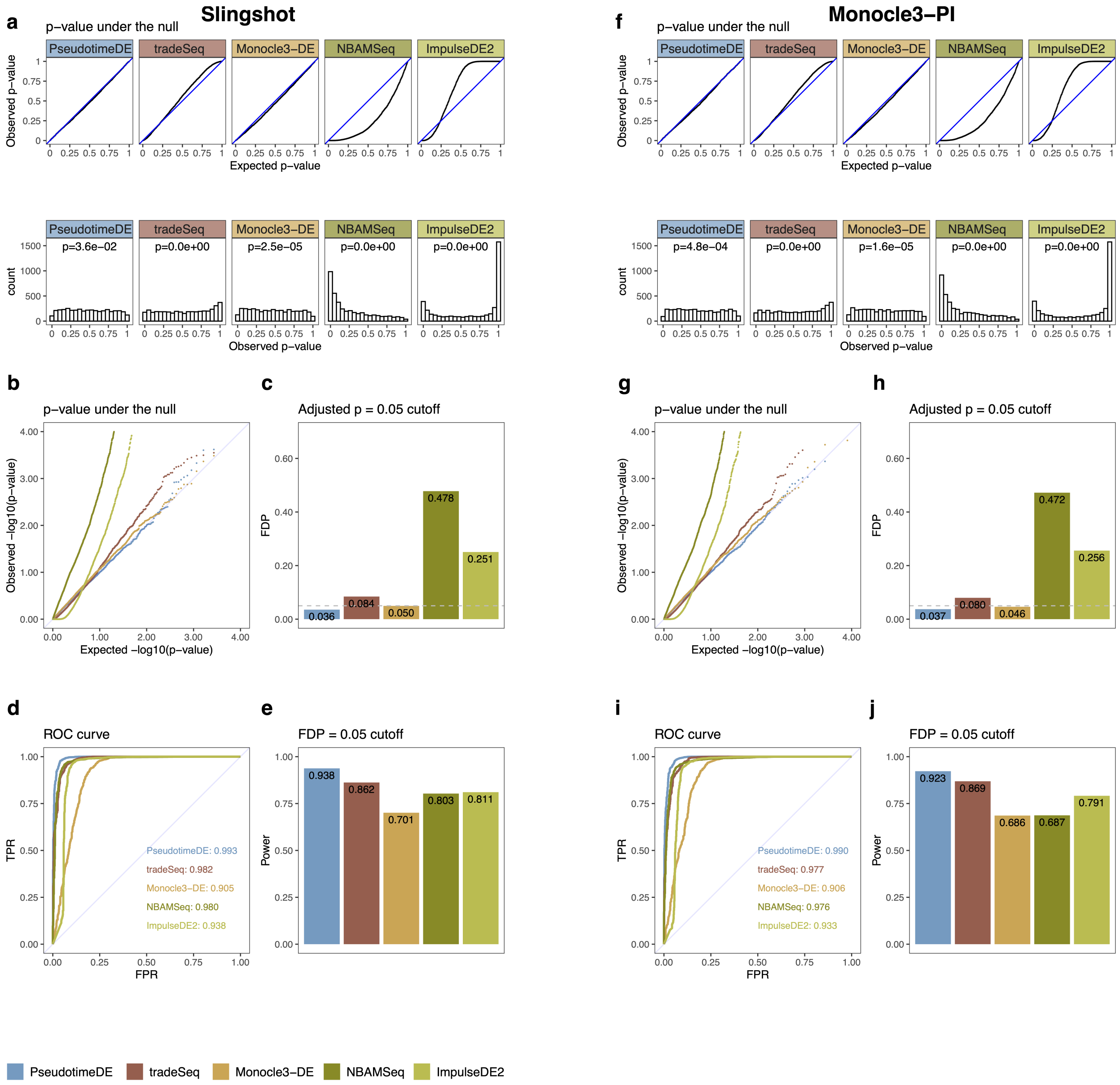

Figure S4: Comparison of five methods (PseudotimeDE, tradeSeq, Monocle3-DE, NBAMSeq, ImpulseDE2) for identifying DE genes along cell pseudotime on synthetic single-lineage data with median dispersion. Left panels (a)–(e) are based on pseudotime inferred by Slingshot; right panels (f)–(j) are based on pseudotime inferred by Monocle3-PI. **(a) & (f)** Distributions of non-DE genes' observed p-values by five DE methods with inferred pseudotime. Top: quantile-quantile plots that compare the empirical quantiles of the observed p-values against the expected quantiles of the Uniform$[0, 1]$ distribution. Bottom: histograms of the observed p-values. The p-values shown on top of histograms are from the Kolmogorov–Smirnov test under the null hypothesis that the distribution is Uniform$[0, 1]$. The larger the p-value, the more uniform the distribution is. Among the five DE methods, PseudotimeDE's observed p-values follow most closely the expected Uniform$[0, 1]$ distribution. **(b) & (g)** Quantile-quantile plots of the same p-values as in (a) and (f) on the negative $\log_{10}$ scale. PseudotimeDE returns better-calibrated small p-values than the other four methods do. **(c) & (h)** FDPs of the five DE methods with the target FDR 0.05 (BH adjusted-$p \leq 0.05$). PseudotimeDE yields the FDP below 0.05, while other methods do not. **(d) & (i)** ROC curves and AUROC values of the five DE methods. PseudotimeDE achieves the highest AUROC. **(e) & (j)** Power of the five DE methods under the FDP = 0.05 cutoff. PseudotimeDE achieves the highest power.

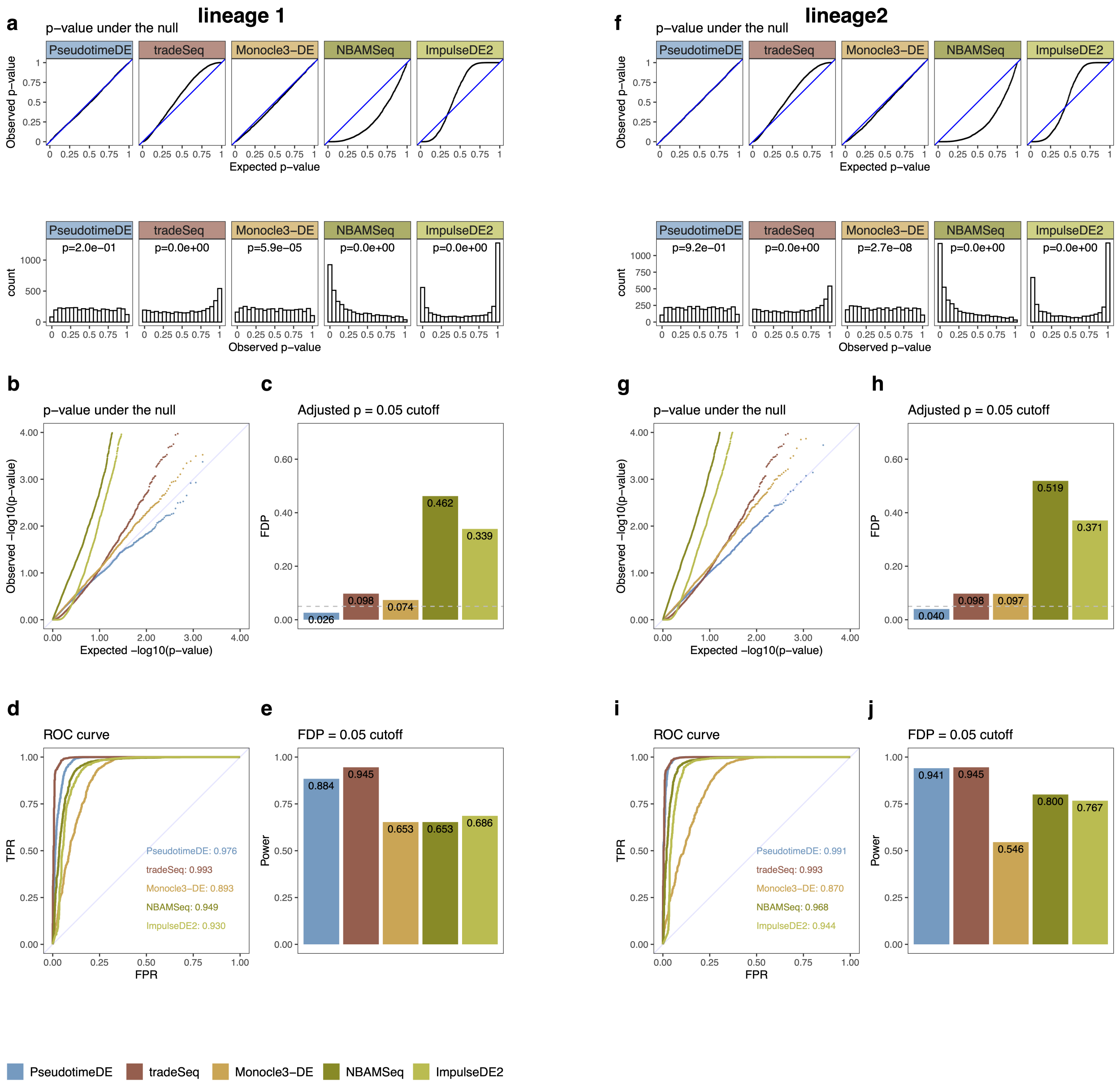

Figure S5: Comparison of five methods (PseudotimeDE, tradeSeq, Monocle3-DE, NBAMSeq, ImpulseDE2) for identifying DE genes along cell pseudotime on synthetic bifurcation data. Pseudotime is inferred by Slingshot. Left panels (a)–(e) are based on lineage 1 of two lineages in bifurcation data; right panels (f)–(j) are based on lineage 2. **(a) & (f)** Distributions of non-DE genes' observed p-values by five DE methods with inferred pseudotime. Top: quantile-quantile plots that compare the empirical quantiles of the observed p-values against the expected quantiles of the Uniform$[0, 1]$ distribution. Bottom: histograms of the observed p-values. The p-values shown on top of histograms are from the Kolmogorov–Smirnov test under the null hypothesis that the distribution is Uniform$[0, 1]$. The larger the p-value, the more uniform the distribution is. Among the five DE methods, PseudotimeDE's observed p-values follow most closely the expected Uniform$[0, 1]$ distribution. **(b) & (g)** Quantile-quantile plots of the same p-values as in (a) and (f) on the negative $\log_{10}$ scale. PseudotimeDE returns better-calibrated small p-values than the other four methods do. **(c) & (h)** FDPs of the five DE methods with the target FDR 0.05 (BH adjusted-$p \leq 0.05$). PseudotimeDE yields the FDP below 0.05, while other methods do not. **(d) & (i)** ROC curves and AUROC values of the five DE methods. PseudotimeDE achieves the second highest AUROC that is close to the highest value. **(e) & (j)** Power of the five DE methods under the FDP = 0.05 cutoff. PseudotimeDE achieves the second highest power that is close to the highest value.

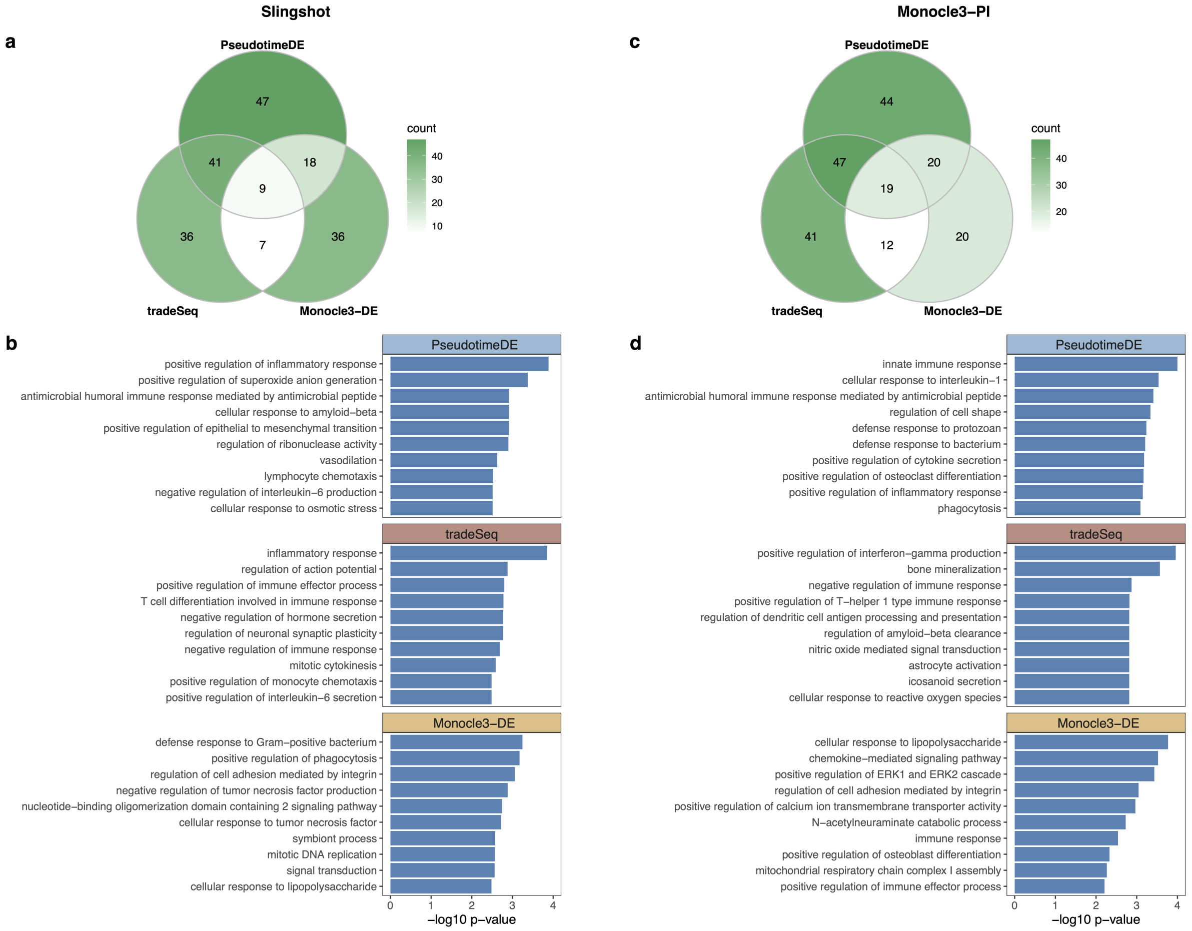

Figure S6: GO analysis of DE genes identified in the LPS-dendritic cell dataset. Left panels (a)–(b) are based on pseudotime inferred by Slingshot; right panels (c)–(d) are based on pseudotime inferred by Monocle3-PI. **(a) & (c)** Numbers of GO terms enriched ($p < 0.01$) in the significant DE genes found by each method in Fig. 4b & f. **(b) & (d)** Top 10 enriched GO terms for each DE method.

a

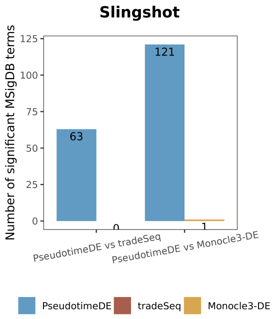

c

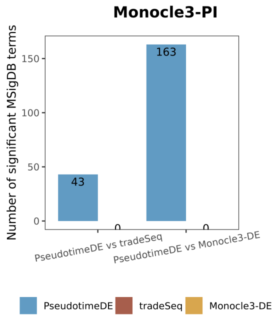

b

PseudotimeDE vs tradeSeq

description	p	p.adj
GSE2706_UNSTIM_VS_2H_LPS_AND_R848_DC_DN	1.2e-05	0.0024
GSE2706_2H_VS_8H_R848_AND_LPS_STIM_DC_DN	2.6e-05	0.0036
GSE14000_4H_VS_16H_LPS_DC_TRANSLATED_RNA_UP	6.4e-05	0.0062

PseudotimeDE vs Monocle3-DE

description	p	p.adj
GSE2706_UNSTIM_VS_2H_LPS_AND_R848_DC_DN	4.1e-07	0.00011
GSE2706_R848_VS_R848_AND_LPS_2H_STIM_DC_DN	3.0e-06	0.00040
GSE14000_UNSTIM_VS_4H_LPS_DC_DN	4.2e-06	0.00051
GSE14000_UNSTIM_VS_4H_LPS_DC_TRANSLATED_RNA_DN	7.1e-06	0.00072
GSE2706_R848_VS_LPS_2H_STIM_DC_DN	5.6e-05	0.00338

d

PseudotimeDE vs tradeSeq

description	p	p.adj
GSE14000_UNSTIM_VS_16H_LPS_DC_TRANSLATED_RNA_UP	4.8e-05	0.0058

PseudotimeDE vs Monocle3-DE

description	p	p.adj
GSE2706_UNSTIM_VS_2H_LPS_DC_DN	7.8e-08	3.1e-05
GSE14000_UNSTIM_VS_4H_LPS_DC_DN	9.9e-07	1.6e-04
GSE2706_UNSTIM_VS_2H_LPS_AND_R848_DC_DN	9.9e-07	1.6e-04
GSE7219_UNSTIM_VS_LPS_AND_ANTI_CD40_STIM_NIK_NFKB2_KO_DC_DN	3.4e-05	2.0e-03
GSE3982_CTRL_VS_LPS_48H_DC_DN	2.4e-04	7.5e-03

Figure S7: MSigDB over-representation analysis of DE genes identified in the LPS-dendritic cell dataset. Left panels (a)–(b) are based on pseudotime inferred by Slingshot; right panels (c)–(d) are based on pseudotime inferred by Monocle3-PI. **(a) & (c)** Numbers of MSigDB terms enriched (BH adjusted-$p < 0.01$) in the significant DE genes specifically found by PseudotimeDE or tradeSeq/Monocle3-DE in pairwise comparisons between PseudotimeDE and tradeSeq/Monocle3-DE in Fig. 4b & f. **(b) & (d)** Example MSigDB terms enriched in the Pseudotime-specific DE genes in (a) & (b). The explanation of terms can be found in MSigDB. All listed terms are related to the response of dendritic cells (DC) stimulated by LPS.

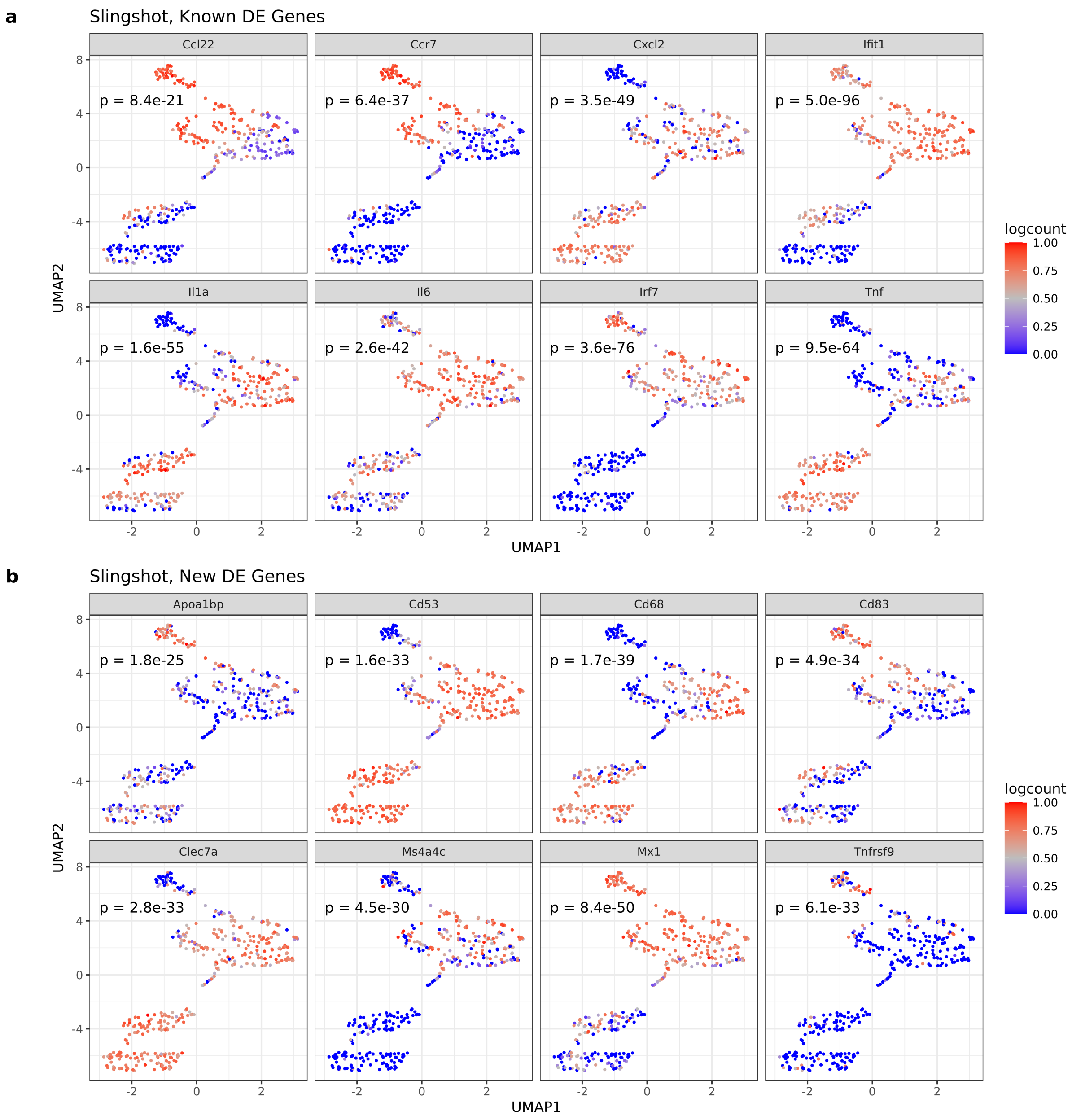

Figure S8: UMAP visualization of example DE genes identified by PseudotimeDE, using Slingshot as the pseudotime inference method, in the LPS-dendritic cell dataset. p-values returned by PseudotimeDE are reported for all the 16 example genes. **(a)** Examples of known DE genes, which have been reported as highly confident DE genes in the original study [7]. **(b)** Examples of new DE genes, which are identified by PseudotimeDE but found as non-DE by either tradeSeq or Monocle3-DE.

a

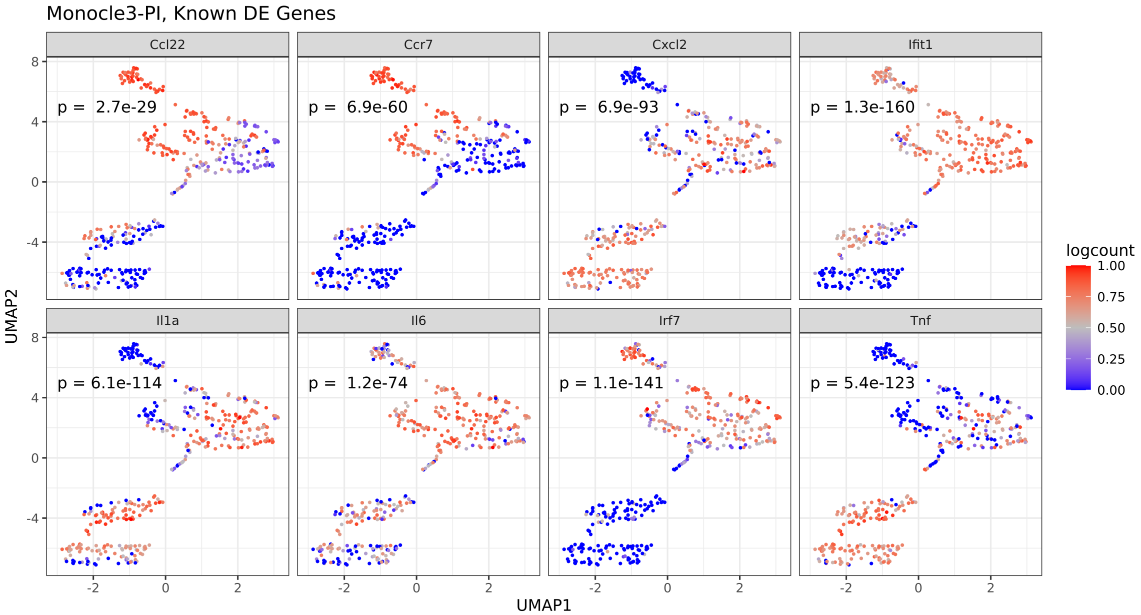

b

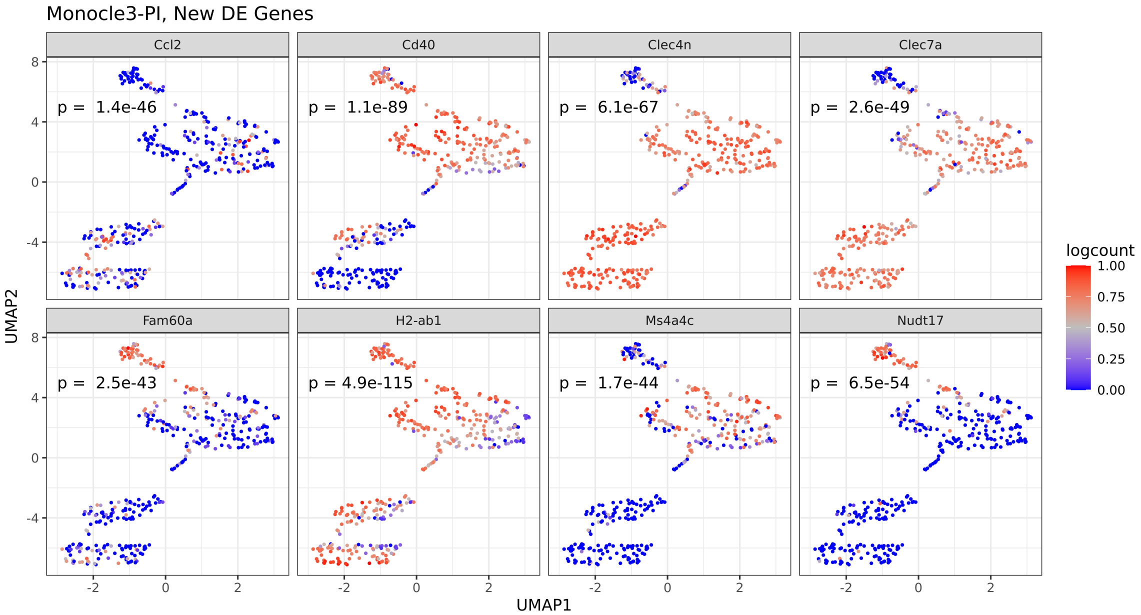

Figure S9: UMAP visualization of example DE genes identified by PseudotimeDE, using Monocle3-PI as the pseudotime inference method, in the LPS-dendritic cell dataset. p-values returned by PseudotimeDE are reported for all the 16 example genes. **(a)** Examples of known DE genes, which have been reported as highly confident DE genes in the original study [7]. **(b)** Examples of new DE genes, which are identified by PseudotimeDE but found as non-DE by either tradeSeq or Monocle3-DE.

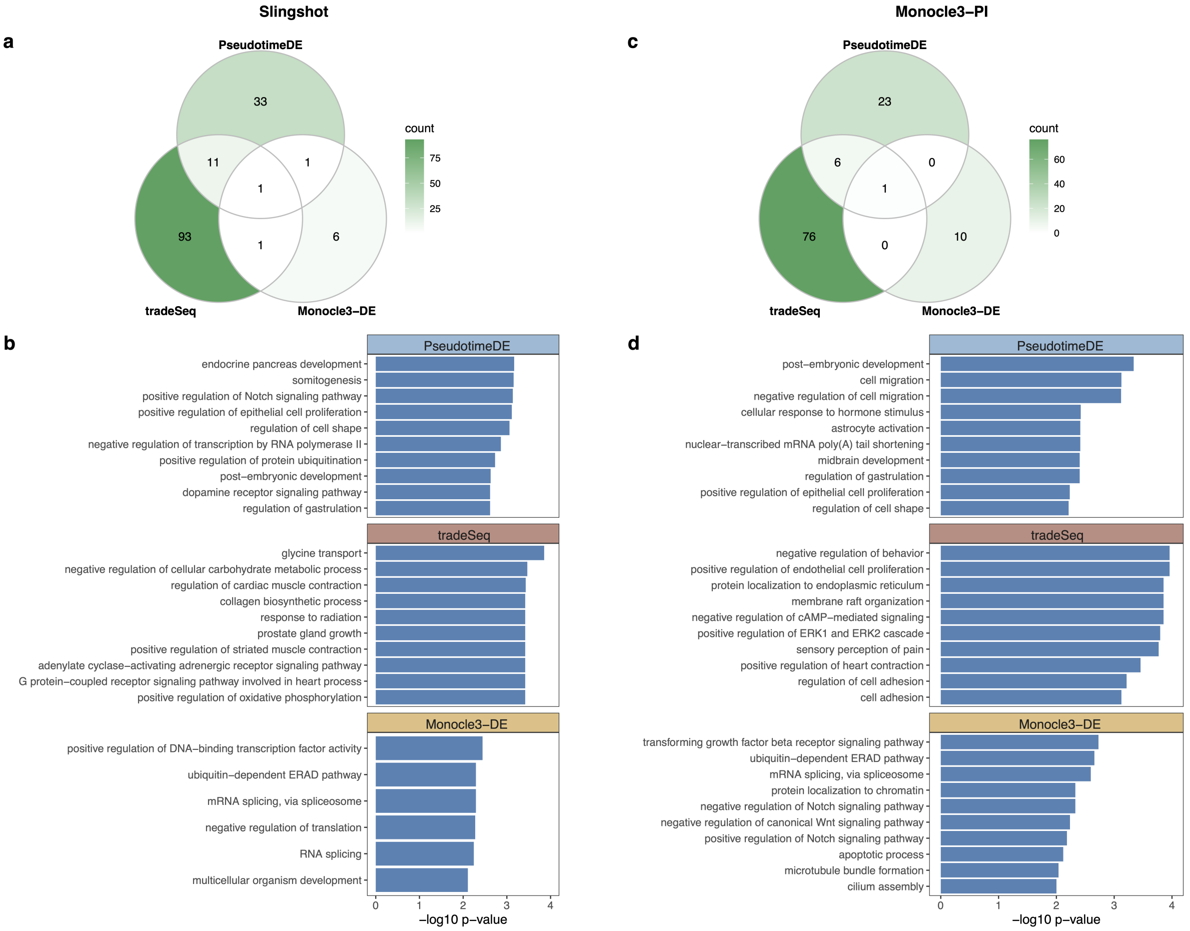

Figure S10: GO analysis of DE genes identified in the pancreatic beta cell maturation dataset. Left panels (a)–(b) are based on pseudotime inferred by Slingshot; right panels (c)–(d) are based on pseudotime inferred by Monocle3-PI. **(a) & (c)** Numbers of GO terms enriched ($p < 0.01$) in the significant DE genes found by each method in Fig. 5b & f. **(b) & (d)** Top 10 significant GO terms for each DE method.

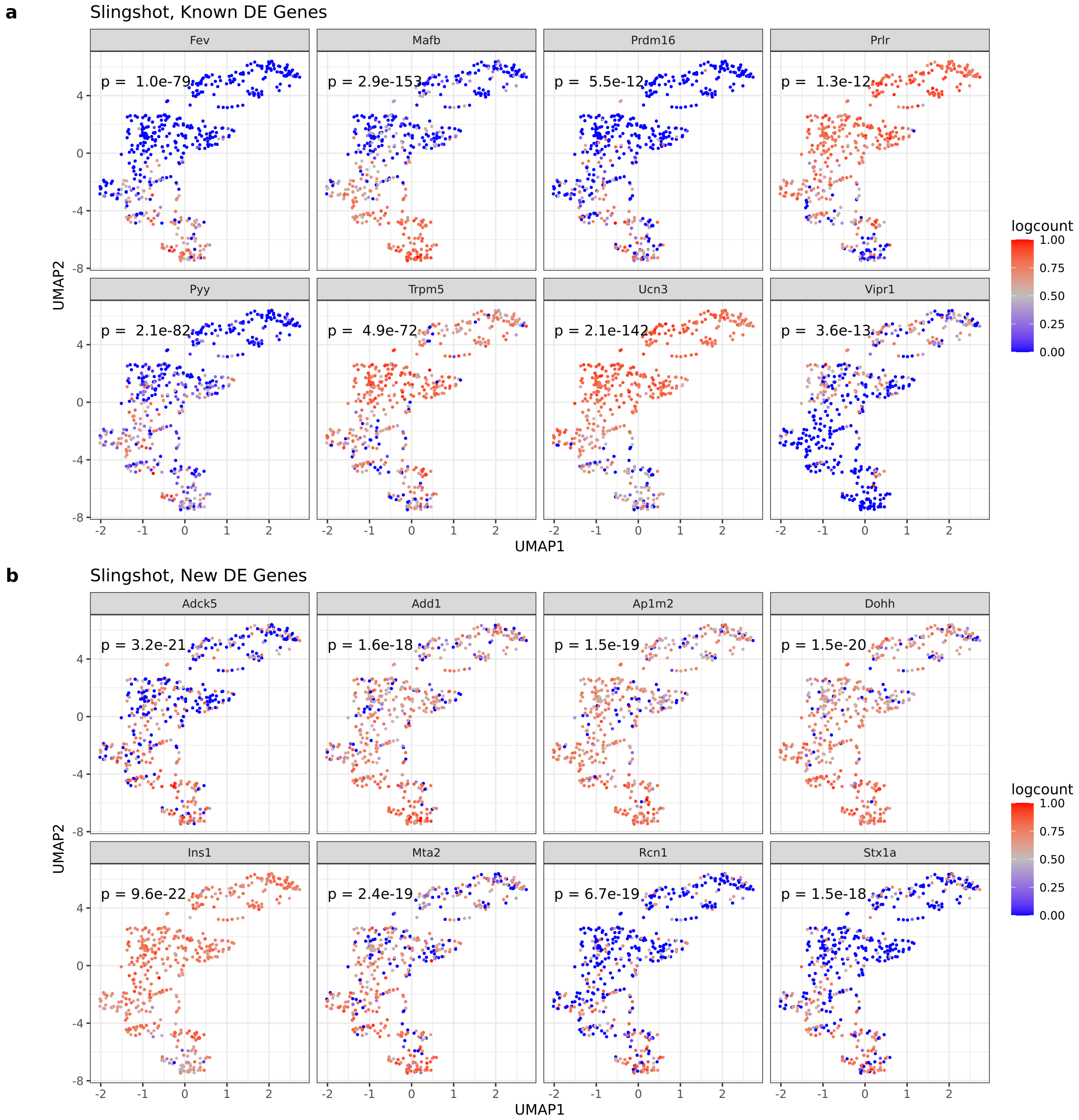

Figure S11: UMAP visualization of example DE genes identified by PseudotimeDE, using Slingshot as the pseudotime inference method, in the pancreatic beta cell maturation cell dataset. p-values returned by PseudotimeDE are reported for all the 16 example genes. **(a)** Examples of known DE genes, which have been reported as highly confident DE genes in the original study [8]. **(b)** Examples of new DE genes, which are identified by PseudotimeDE but found as non-DE by either tradeSeq or Monocle3-DE.

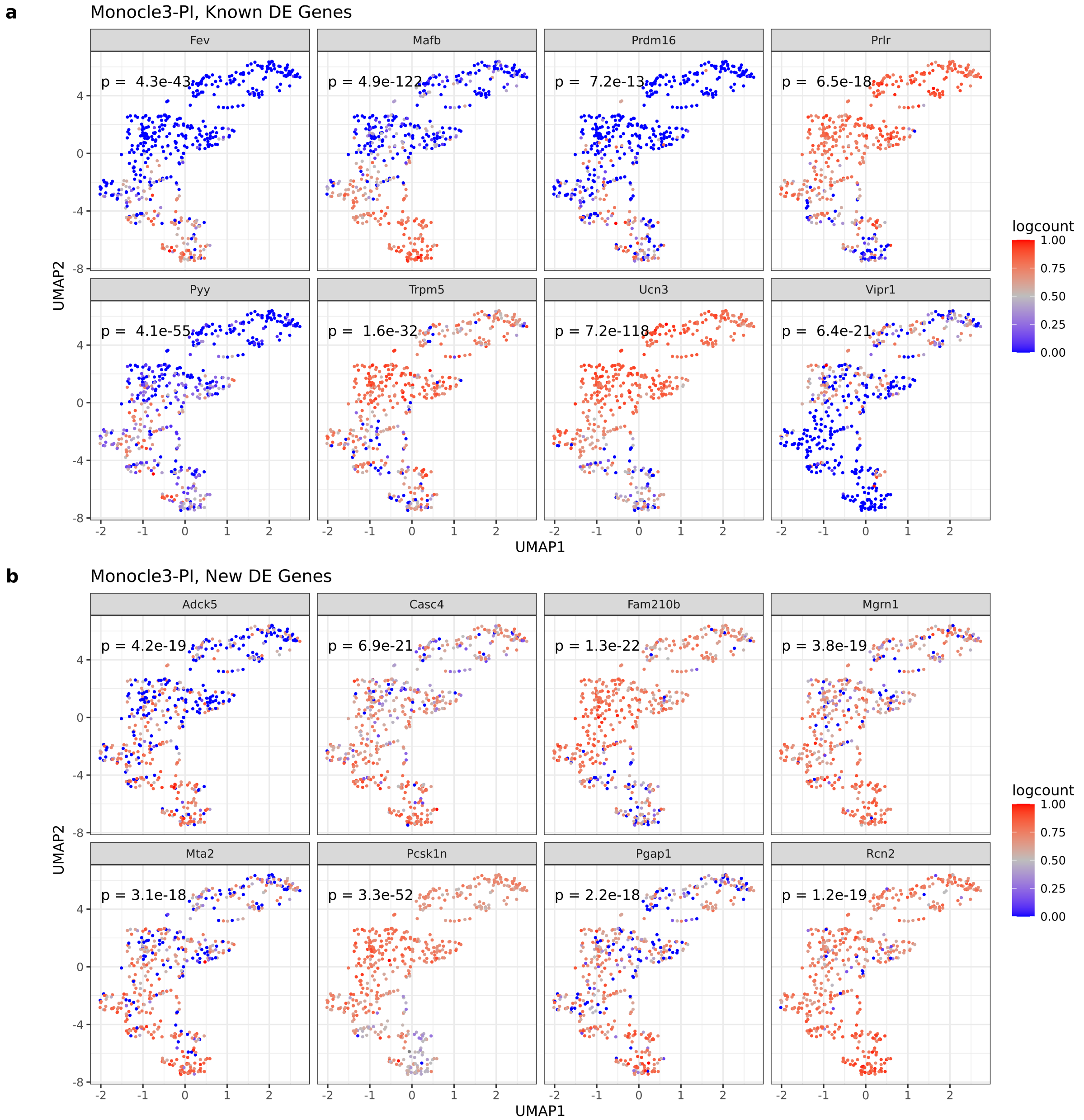

Figure S12: UMAP visualization of example DE genes identified by PseudotimeDE, using Monocle3-PI as the pseudotime inference method, in the pancreatic beta cell maturation cell dataset. p-values returned by PseudotimeDE are reported for all the 16 example genes. **(a)** Examples of known DE genes, which have been reported as highly confident DE genes in the original study [8]. **(b)** Examples of new DE genes, which are identified by PseudotimeDE but found as non-DE by either tradeSeq or Monocle3-DE.

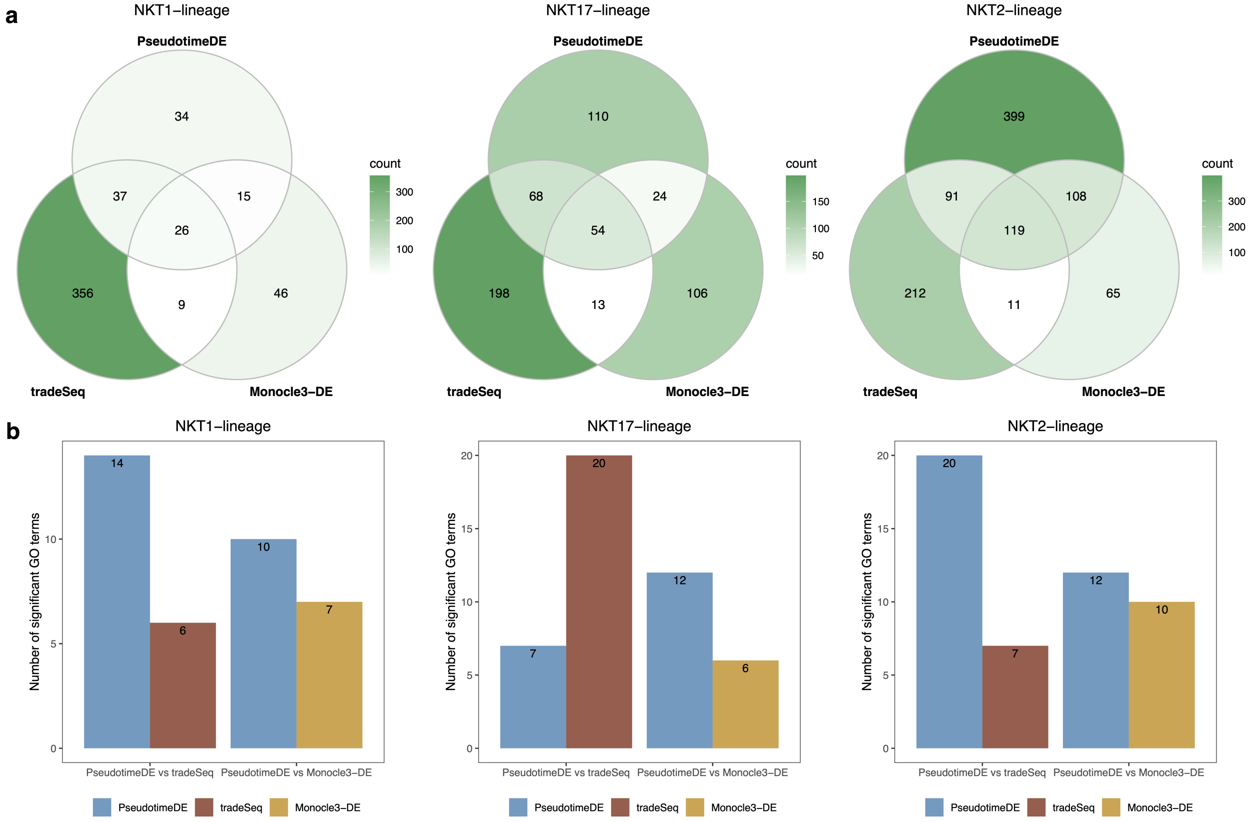

Figure S13: GO analysis of DE genes identified in the natural killer T cell dataset. (a) Venn plots showing the overlaps of the significant DE genes (BH adjusted-$p \leq 0.05$) identified by the three DE methods. **(b)** Numbers of GO terms enriched ($p < 0.05$) in the significant DE genes specifically found by PseudotimeDE or tradeSeq/Monocle3-DE in pairwise comparisons between PseudotimeDE and tradeSeq/Monocle3-DE in (a).

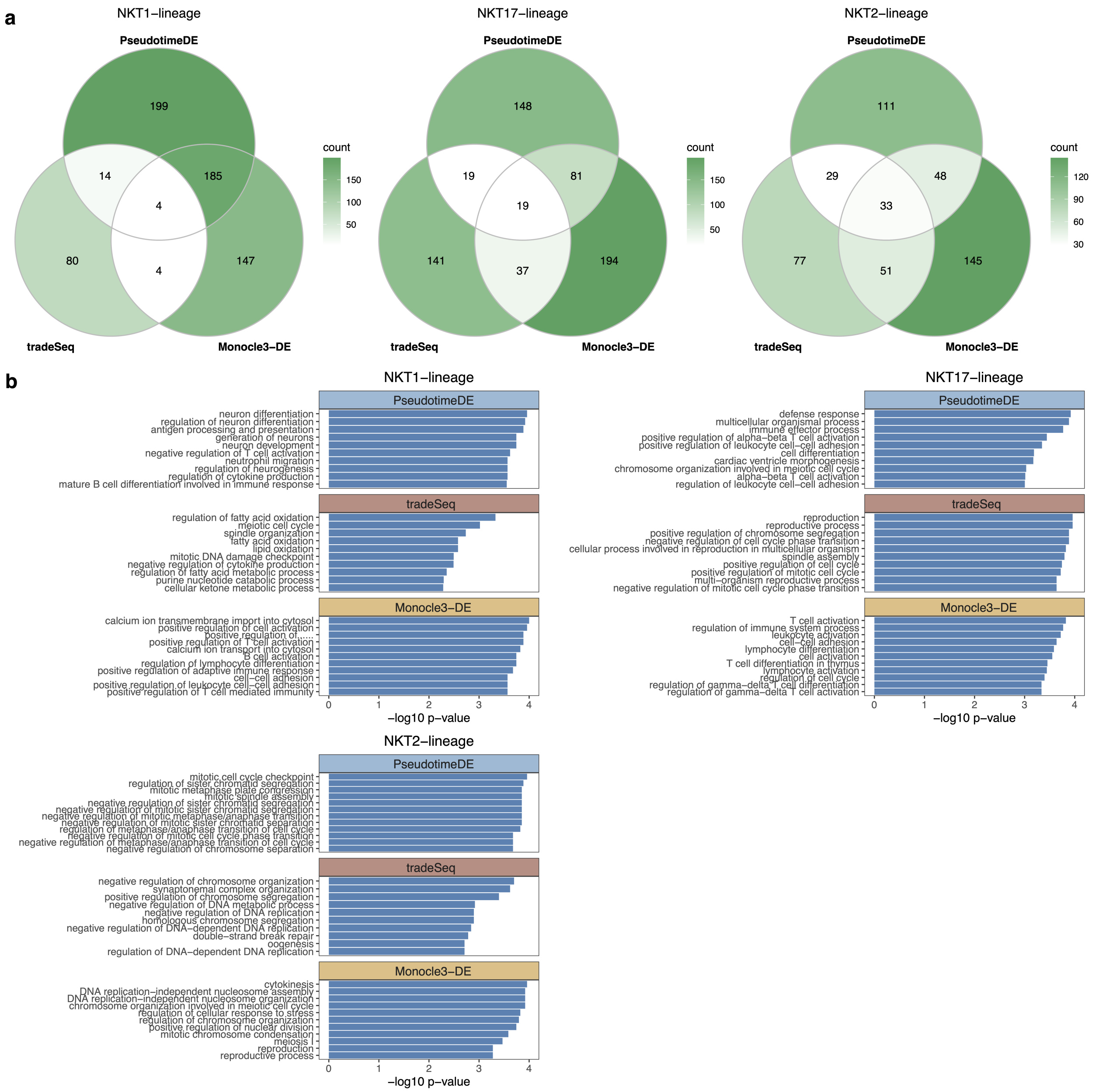

Figure S14: GO analysis of DE genes identified in the natural killer T cell dataset. **(a)** Numbers of GO terms enriched ($p < 0.05$) in the significant DE genes found by each method. **(b)** Top 10 enriched GO terms for each DE method.

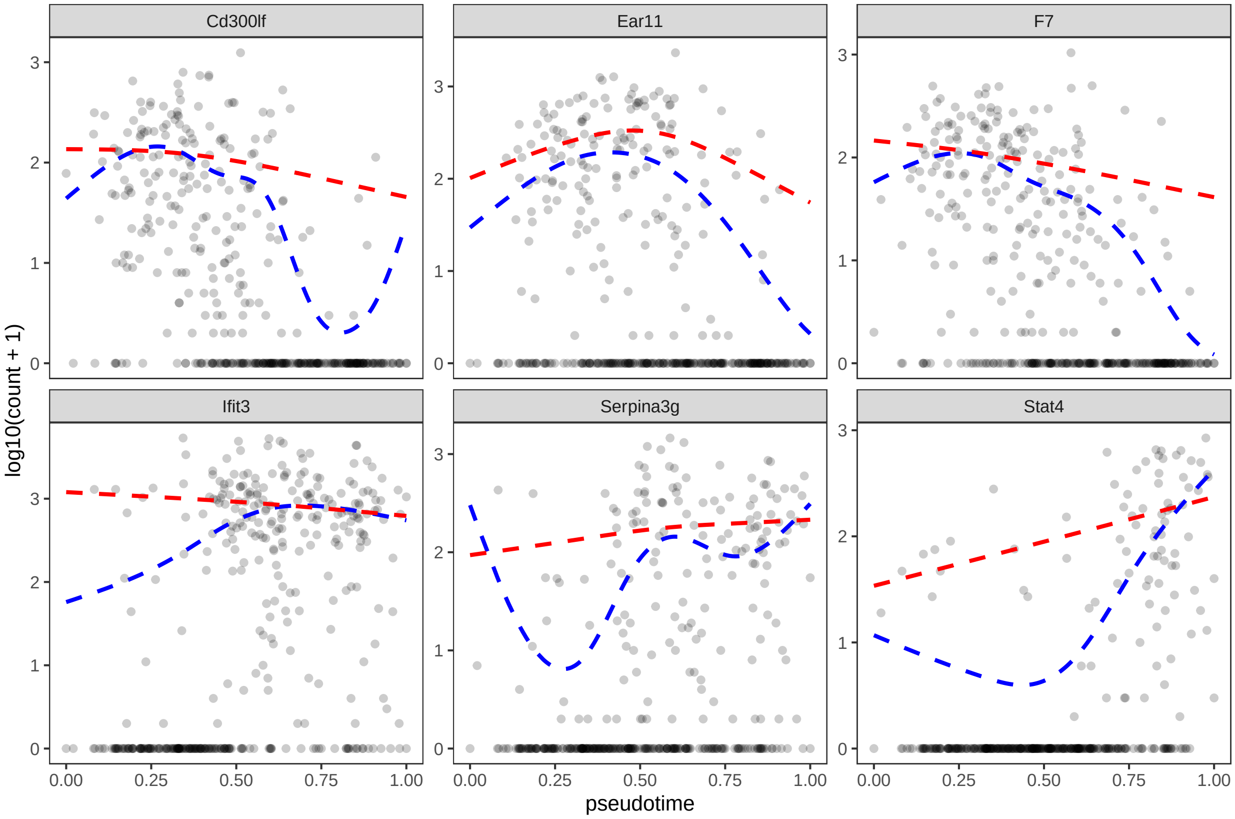

Figure S15: Comparison of NB-GAM and ZINB-GAM on the LPS-dendritic cell dataset with Slingshot pseudotime. Example fitted results of NB-GAM / ZINB-GAM on six genes from the LPS-dendritic cell dataset with pseudotime inferred by Slingshot. NB-GAM yields small p-values ($p < 1\text{e}-10$) and ZINB-GAM yields large p-values ($p > 0.01$). Dashed blue lines and red lines are the fitted curves by NB-GAM and ZINB-GAM, respectively.

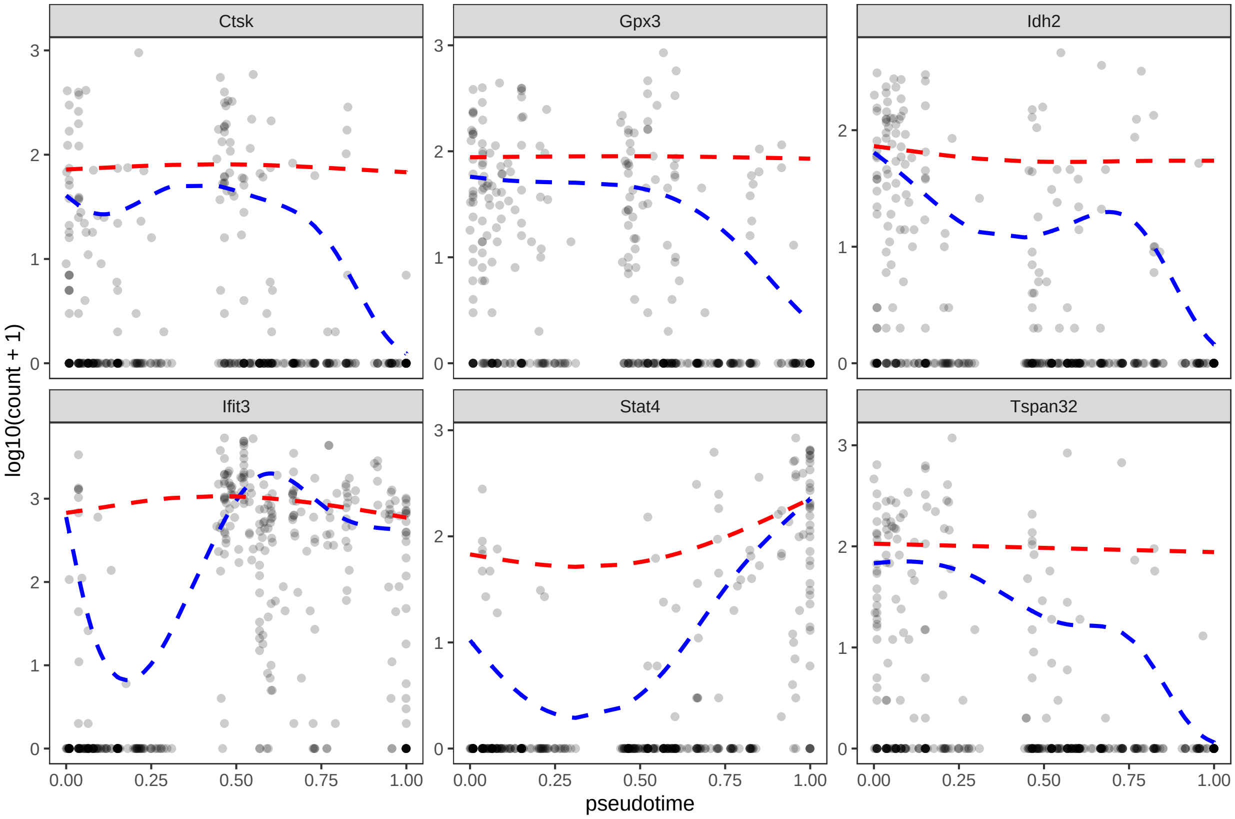

Figure S16: Comparison of NB-GAM and ZINB-GAM on the LPS-dendritic cell dataset with Monocle3-PI pseudotime. Example fitted results of NB-GAM / ZINB-GAM on six genes from the LPS-dendritic cell dataset with pseudotime inferred by Monocle3-PI. NB-GAM yields small p-values ($p < 1\mathrm{e}-10$) and ZINB-GAM yields large p-values ($p > 0.01$). Dashed blue lines and red lines are the fitted curves by NB-GAM and ZINB-GAM, respectively.

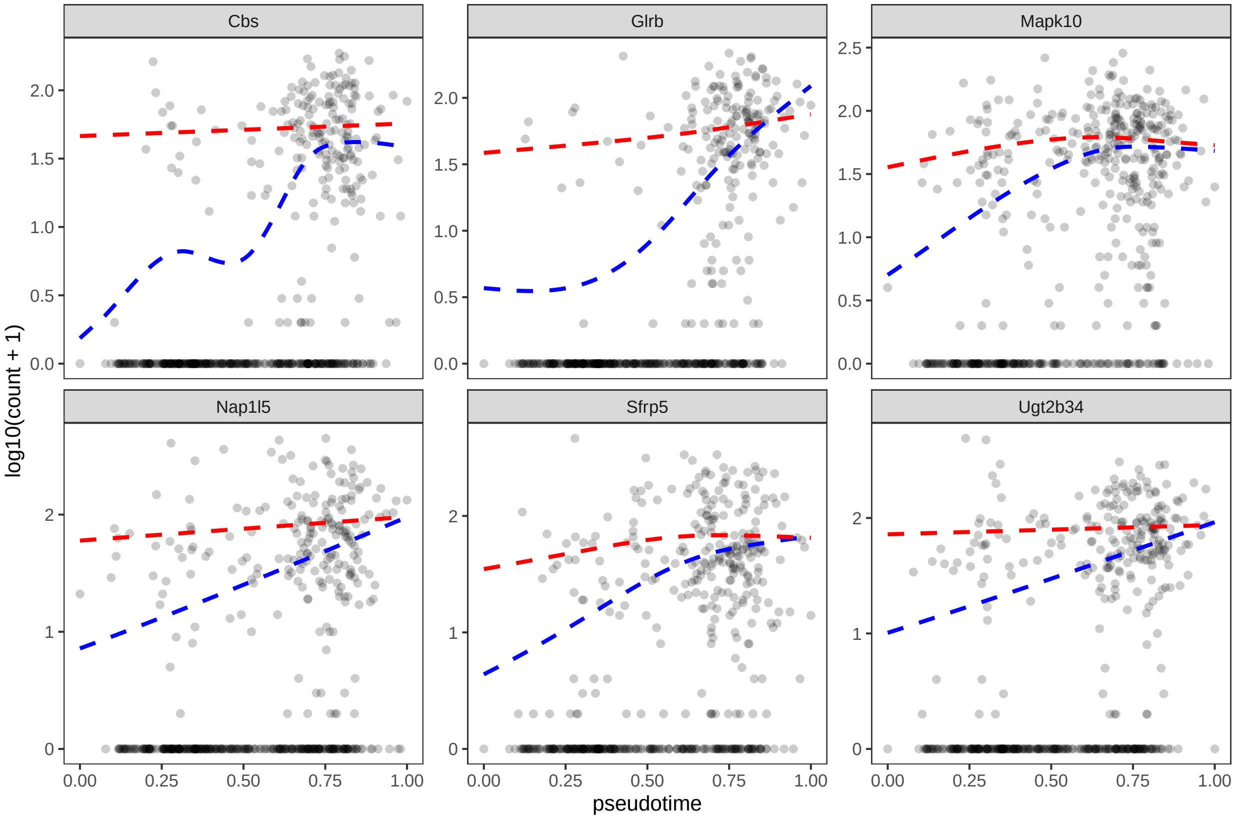

Figure S17: Comparison of NB-GAM and ZINB-GAM on the pancreatic beta cell maturation dataset with Slingshot pseudotime. Example fitted results of NB-GAM / ZINB-GAM on six genes fromthe pancreatic beta cell maturation dataset with pseudotime inferred by Slingshot. NB-GAM yields small p-values ($p < 1\mathrm{e}-10$) and ZINB-GAM yields large p-values ($p > 0.01$). Dashed blue lines and red lines are the fitted curves by NB-GAM and ZINB-GAM, respectively.

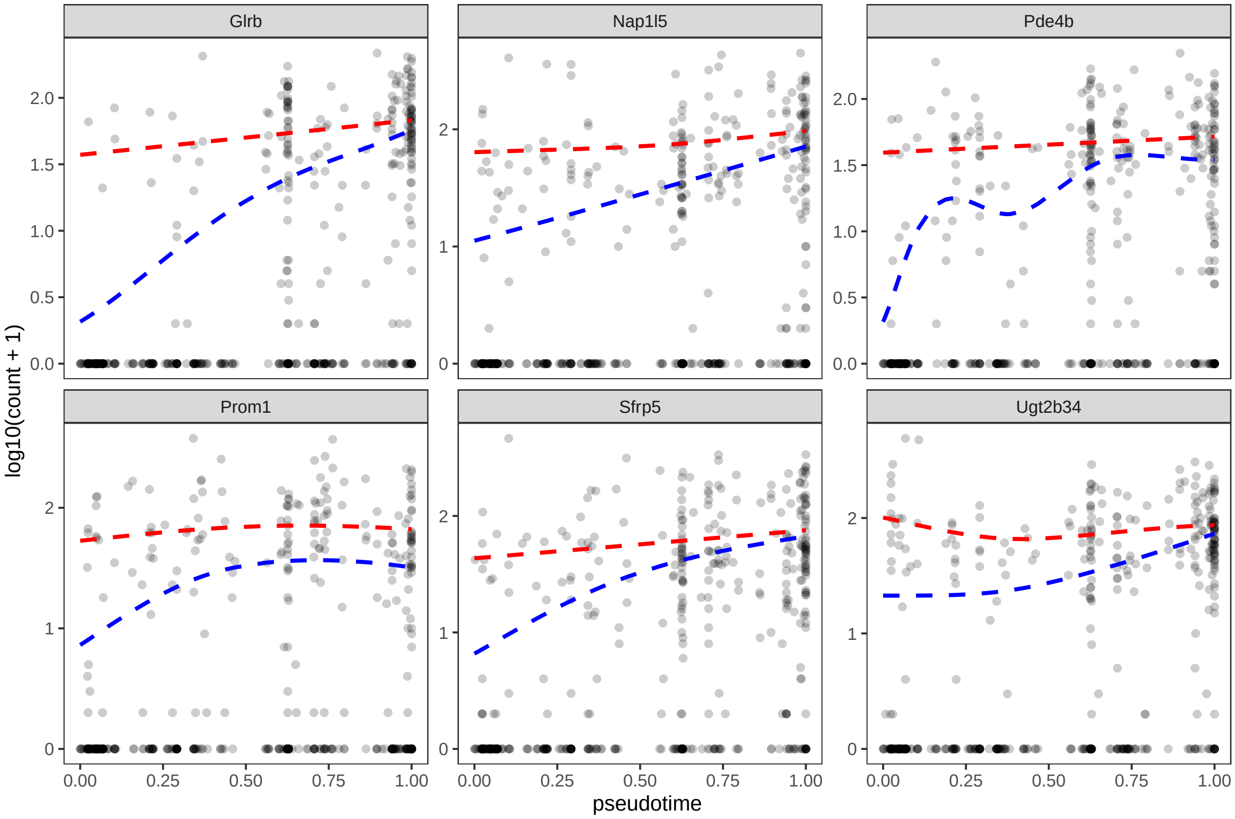

Figure S18: Comparison of NB-GAM and ZINB-GAM on the pancreatic beta cell maturation cell dataset with Slingshot pseudotime. Example fitted results of NB-GAM / ZINB-GAM on six genes from the pancreatic beta cell maturation with pseudotime inferred by Monocle3-PI. NB-GAM yields small p-values ($p < 1\mathrm{e}-10$) and ZINB-GAM yields large p-values ($p > 0.01$). Dashed blue lines and red lines are the fitted curves by NB-GAM and ZINB-GAM, respectively.

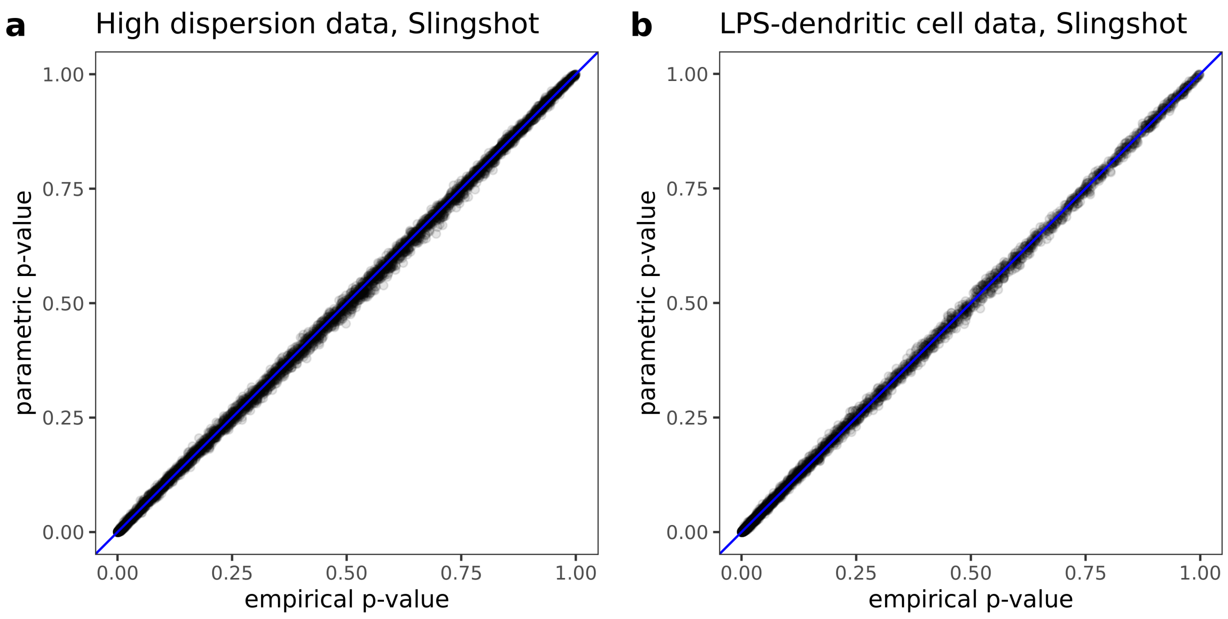

Figure S19: Comparison of empirical p-value and parametric p-value. Scatter plot of empirical p-values and parametric p-values (see **Methods**). **(a)** Scatter plot based on synthetic high dispersion dataset and pseudotime inferred by Slingshot. **(b)** Scatter plot based on LPS-dendritic cell dataset and pseudotime inferred by Slingshot. The parametric p-values are perfectly correlated with empirical p-values, suggesting that the parametric model well captures the estimated null distribution of test statistics.

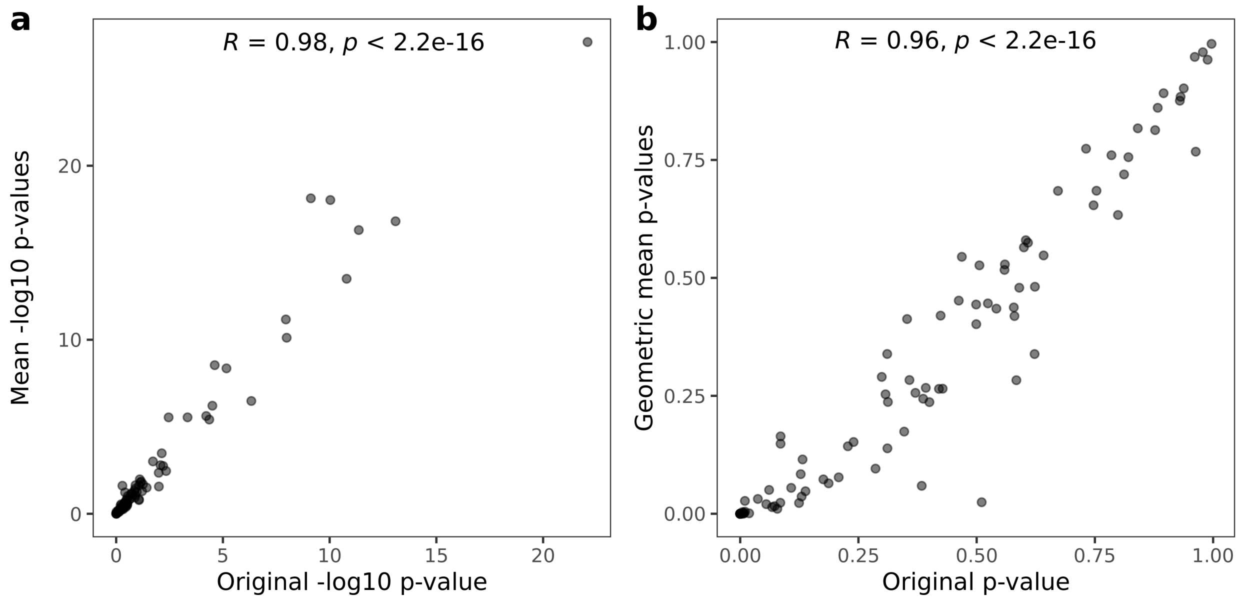

Figure S20: Comparison of p-values using 1000 subsamples and p-values using 100 subsamples. The p-values are based on based on synthetic high dispersion dataset and pseudotime inferred by Slingshot. **(a)** Scatter plot of the original p-value using 1000 subsamples on negative $-\log_{10}$ scale and the mean of 50 p-values using 100 subsamples on negative $-\log_{10}$ scale. The strict linearity (Pearson correlation coefficient $R = 0.98$) suggests that using 100 subsamples yield similar p-values to those of using 1000 subsamples on the negative log scale. **(b)** Scatter plot of the original p-value using 1000 subsamples and the geometric mean of 50 p-values using 100 subsamples. The strict linearity (Pearson correlation coefficient $R = 0.96$) suggests that using 100 subsamples yield similar p-values to those of using 1000 subsamples on the original scale.

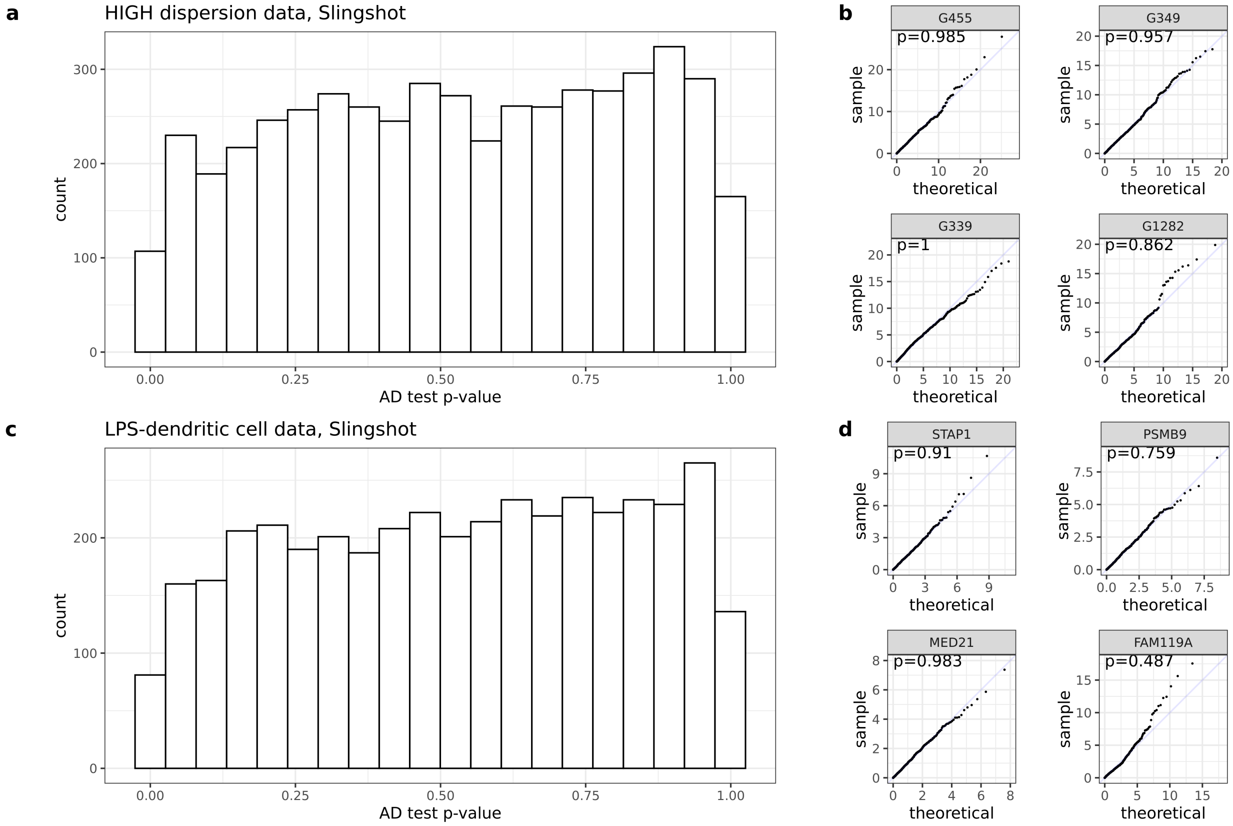

Figure S21: Goodness-of-fit of the parametric distribution. The p-values are from the Anderson-Darling (AD) test, which measures the goodness-of-fit of the gamma/two-component gamma mixture distribution to the empirical null distribution generated by subsampling and permutation. **(a)** Histogram of AD test p-values based on the synthetic high dispersion dataset and pseudotime inferred by Slingshot. The distribution is approximately Uniform$[0, 1]$, indicating that the parametric distribution fits the empirical null distribution well. **(b)** Quantile-quantile plots comparing the empirical null distribution and its corresponding parametric fit for four random genes. **(c)** Histogram of AD test p-values based on the LPS-dendritic cell dataset and pseudotime inferred by Slingshot. The distribution is approximately Uniform$[0, 1]$, indicating that the parametric distribution fits the empirical null distribution well. **(d)** Quantile-quantile plots comparing the empirical null distribution and its corresponding parametric fit for four random genes.

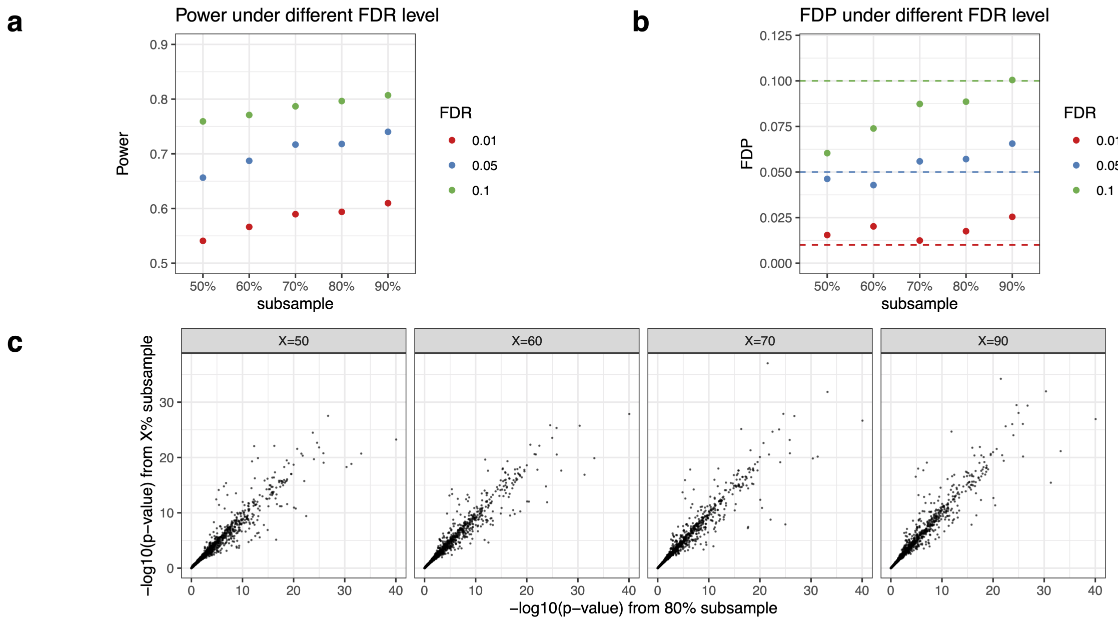

Figure S22: Robustness of PseudotimeDE to the subsampling proportion. Results are based on the synthetic high dispersion dataset and pseudotime inferred by Slingshot. **(a)** Power of PseudotimeDE using different subsampling proportions under FDR levels 0.01, 0.05, and 0.1. **(b)** FDP of PseudotimeDE using different subsampling proportions under FDR levels 0.01, 0.05, and 0.1. **(c)** Scatter plots of the default p-values using 80% as the subsampling proportion vs. the p-values using 50%, 60%, 70% or 90% as the subsampling proportion. The strong linearity (Pearson correlation coefficient $R \geq 0.96$) of p-values under different subsampling proportions confirms the robustness of PseudotimeDE to the subsampling proportion.

References

[1] Michael I Love, Wolfgang Huber, and Simon Anders. Moderated estimation of fold change and dispersion for rna-seq data with deseq2. *Genome biology*, 15(12):550, 2014.

[2] Davide Risso, Fanny Perraudeau, Svetlana Gribkova, Sandrine Dudoit, and Jean-Philippe Vert. A general and flexible method for signal extraction from single-cell rna-seq data. *Nature communications*, 9(1):1–17, 2018.

[3] Simon N. Wood. Basis dimension choice for smooths. URL `https://stat.ethz.ch/R-manual/R-patched/library/mgcv/html/choose.k.html`.

[4] Koen Van den Berge, Hector Roux De Bezieux, Kelly Street, Wouter Saelens, Robrecht Cannoodt, Yvan Saeys, Sandrine Dudoit, and Lieven Clement. Trajectory-based differential expression analysis for single-cell sequencing data. *Nature communications*, 11(1):1–13, 2020.

[5] Simon N Wood. *Generalized additive models: an introduction with R*. CRC press, 2017.

[6] Simon N Wood. On p-values for smooth components of an extended generalized additive model. *Biometrika*, 100(1):221–228, 2013.

[7] Alex K Shalek, Rahul Satija, Joe Shuga, John J Trombetta, Dave Gennert, Diana Lu, Peilin Chen, Rona S Gertner, Jellert T Gaublomme, Nir Yosef, et al. Single-cell rna-seq reveals dynamic paracrine control of cellular variation. *Nature*, 510(7505):363–369, 2014.

[8] Wei-Lin Qiu, Yu-Wei Zhang, Ye Feng, Lin-Chen Li, Liu Yang, and Cheng-Ran Xu. Deciphering pancreatic islet β cell and α cell maturation pathways and characteristic features at the single-cell level. *Cell metabolism*, 25(5):1194–1205, 2017.
