# Supplementary material for: PseudotimeDE: inference of differential gene expression along cell pseudotime with well-calibrated p-values from single-cell RNA sequencing data
Source: Genome Biol. 2021 Apr 29;22:124. doi: 10.1186/s13059-021-02341-y (PMC8082818; doi:10.1186/s13059-021-02341-y)
Supplement: Supplementary file 5 — Additional file 5 Review history. [file 13059_2021_2341_MOESM5_ESM.docx]

Review History

**First round of review**

**Reviewer 1**

**Are you able to assess all statistics in the manuscript, including the appropriateness of statistical tests used?**

Yes: Please refer to the comments to the reviewers

**Comments to author:**

Reviewer comment for "PseudotimeDE: inference of differential gene expression along cell pseudotime with valid p-values from single-cell RNA sequencing data"

Trajectory/pseudotime inference and the subsequent inference of differential gene expression along the inferred pseudotime are important aspects in analyzing single-cell genomic data. Because of the noisiness nature in single-cell datasets, there is high uncertainty in inferring the pseudotime, and most existing DE methods do not take into account this uncertainty in inferring the DE genes. In this work, the authors provide an approach based on subsampling, permutation, and negative binomial (NB)/zero-inflated NB generalized additive models. They showed that ignoring the uncertainty in pseudotime inference can lead to loss of power. Through simulations and three real data examples, they demonstrated that their proposed method can control the false discovery rate and has high power versus the benchmarked methods. The evaluation and comparisons with other methods looks solid and convincing. The manuscript is very well-written and it is likely of significance to the field of single-cell genomics. Here are my comments:

1. In the subsampling step, PseudotimeDE subsamples 80% of cells from the original dataset. How rigorous is the performance when the proportion of subsampling varies?

2. To estimate the null distribution, the authors implemented one component and two component Gamma mixture models. How good is the fit?

3. In figure 3a and f, the null distributions for tradeSeq look quite different, while the distributions look similar in all other methods. Is there any possible explanation for this observation?

4. As the authors mentioned in the introduction, some other methods (e.g. tradeSeq) also implemented NB-GAM for DE inference. For model fitting of NB-GAM, what is the difference between PseudotimeDE and other mentioned methods? It would be great if the authors can provide some discussion.

5. On the Github page for the software, only the Rmd vignette file is provided. It would be good to provide Html/pdf vignettes.

6. Typo in figure 3: "FDP" -> "FDR

**Reviewer 2**

**Are you able to assess all statistics in the manuscript, including the appropriateness of statistical tests used?**

Yes: Additional review not needed.

**Comments to author:**

In this manuscript by Song and Li, the authors describe PseudotimeDE, a method that accounts for psuedotime inference uncertainty from single-cell RNA-seq (scRNA-seq) data and computes differential expressed (DE) genes with calibrated p-values. The work tries to address the concern that typical pseudotime inference (PI) approaches return point estimates without uncertainty quantification/standard error, leading to either invalid/poor FDR-controlled p-values for downstream analysis incl. DE. PseudotimeDE works by first inferring pseudotime on subsample data using slingshot, Monocle, alongside user input original pseudotime, followed by model fitting using NBGAM or ZINB-GAM. Generating a null distribution by fitting a gamma (mixture of gamma) distribution to non-DE genes from permuted subsamples, the method finds DE genes with calibrated, FDR-controlled p-values.

The authors first apply their approach in simulated datasets with varying levels of expression dispersions on single-lineage dataset and a bifurcation dataset. This includes assessing pseudotime inference uncertainty (Using slingshot or Monocle3) on subsampled dataset, and identifying, benchmarking DE genes against 4 methods (tradeSeq, monocle3, NBAMSeq and ImpulseDE2). Utilizing Slingshot and monocle inferred pseudotime on subsamples, alongside to single-point inference estimates from other approaches, the authors PseudotimeDE fares better in DE genes with calibrated p-values in different comparisons (distribution of non-DE genes, false discovery proportions/FDP, AUROC, power under 0.05 FDP). The authors apply their approach on 3 published scRNA-seq datasets including mouse dendritic cell stimulation by LPS, mouse pancreatic beta cell differentiation and mouse bone marrow differentiation; and compare DE genes computed by tradeSeq, Monocle3 and PseudotimeDE. Applying both inferences (slingshot & monocle3) on dendritic cell data, the authors find most DE genes shared between all methods; notably with significant subset of DE genes found by monocle3 and PseudotimeDE alone; and robust, meaningful GO terms and enriched molecular signature terms. Similarly the authors compare, identify and highlight DE genes with calibrated p-values in beta-cell differentiation data, particularly demarcating Slc39a10 as a DE gene and somatostatin as a non-DE gene. Applying to a multi-trajectory bone marrow dataset, the authors find PseudotimeDE captures different subset of DE genes and p-values that authors highlight as biologically relevant by GSEA analysis. Lastly the authors report on higher PseudotimeDE computation times owing to subsampling and permutations, including potential speed up strategies (limited subsampling, filtering zero-count/low quality genes, fixed pseudotime).

The approach is well designed, catering to a important question in single-cell analysis and method would be potentially useful to the community. There are a few concerns stated below

-	It is critical that the authors demonstrate their approach on additional real/published datasets with multiple trajectories and/or lineages.

-	While the authors leave the choice of topology/lineage to users, it would be beneficial to further demonstrate suitability of PseudotimeDE to cyclic, linear, converging/binary real datasets

-	The authors should also show a few examples of new/predicted DE genes from analysed datasets in a tSNE/UMAP to visually assess heterogeneity between dropouts and expressed single-cells.

-	In Fig 4, its unclear how there are more exclusive GO terms captured by PseudotimeDE (195 unique DE genes) in comparison to Monocle3 (172 unique DE genes)

-	Typos (monolce3, Nap1|5)

**Authors Response**

**Point-by-point responses to the reviewers’ comments:**

*We thank the two reviewers for their constructive comments. We have addressed all of them and modified the paper accordingly. Our detailed point-by-point answers are in the following pages.*

Answers to Reviewer 1

In this work, the authors provide an approach based on subsampling, permutation, and negative binomial (NB)/zero-inflated NB generalized additive models. They showed that ignoring the uncertainty in pseudotime inference can lead to loss of power. Through simulations and three real data examples, they demonstrated that their proposed method can control the false discovery rate and has high power versus the benchmarked methods. The evaluation and comparisons with other methods looks solid and convincing. The manuscript is very well-written and it is likely of significance to the field of single-cell genomics.

*We thank the reviewer for appreciating the merit of our work. Here are my comments:*

Comment R1.1 In the subsampling step, PseudotimeDE subsamples 80% of cells from the original dataset. How rigorous is the performance when the proportion of subsampling varies?

*Answer to R1.1 We thank the reviewer for this important comment. In general, we find the results of PseudotimeDE robust to the subsampling proportion. In our revision, we have applied to a simulation dataset (Fig. R1) five sampling proportions (50%, 60%, 70%, 80%, and 90%), among which 80% is the default used in PseudotimeDE, as the reviewer noted. From the results, we first observe that the -values (on the − log10 scale) are highly correlated between 80% and any other proportion (Fig. R1c), confirming the robustness of PseudotimeDE to the subsampling proportion. Second, we observe that the power of PseudotimeDE remains stable under varying sampling proportions, though the power is slightly lower when the sampling proportion is 50% (Fig. R1a). Third, the false discovery proportion (FDP) of PseudotimeDE is also stable under varying sampling proportions, though the FDP is slightly higher than the nominal level when the sampling proportion is 90% (Fig. R1b). Therefore, we conclude that 80% is a reasonable default sampling proportion. We have added Fig. R1 to the revised manuscript as Fig. S22.*

Comment R1.2 To estimate the null distribution, the authors implemented one component and two component Gamma mixture models. How good is the fit?

*Answer to R1.2 We thank the reviewer for raising this important question. We perform a goodness-of-fit test to show that our parametric approach fits the empirical distributions well (Fig. R2). We choose the Anderson-Darling (AD) test since it gives more weights on the tail of the distribution than the Kolmogorov-Smirnov test does. On one simulated dataset and one real dataset, the distribution of AD test p-values is approximately Uniform[0, 1] (Fig. R2a & c), indicating that the parametric distributions fit the empirical null distributions well (i.e., all cases are from the null). Realizing this goodness-of-fit check is crucial, we have revised our PseudotimeDE R pack¬age to output the AD test p-value so that users can check whether the parametric approximation works well; if not, users may increase the number of subsampling and use the empirical null for DE p-value calculation.*

*Figure R1: Robustness*

*PseudotimeDE to the subsampling proportion.Results are based on the synthetic high dispersion dataset and pseudotime inferred by Slingshot. (a) Power of PseudotimeDE using different subsampling proportions under FDR levels 0.01, 0.05, and 0.1. (b) FDP of PseudotimeDE using different subsampling proportions under FDR levels 0.01, 0.05, and 0.1. (c) Scatter plots of the default -values using 80% as the subsampling proportion vs. the -values using 50%, 60%, 70% or 90% as the subsampling proportion. The strong linearity (Pearson correlation coefficient ≥ 096) of -values under different subsampling proportions confirms the robustness of PseudotimeDE to the subsampling proportion.*

Comment R1.3 In figure 3a and f, the null distributions for tradeSeq look quite different, while the distributions look similar in all other methods. Is there any possible explanation for this observation?

*Answer to R1.3*

*The reason for this dramatic difference is tradeSeq’s (1) sensitivity to the number of knots and (2) incorrect null distribution. The number of knots, K, a parameter in the generalized additive model (GAM), defines the number and positions of piecewise polynomial functions in a spline function.*

*where fj(•) is the smooth spline function of gene j, Ti is the pseudotime of cell i, and K is the number of knots. It is worth noting that K − 1 is the nominal degree of freedom of the model.*

*K needs to be specified before the negative binomial-GAM (NB-GAM) is fitted. To choose K, tradeSeq randomly samples a small set of genes and uses AIC to find an “appropriate” K for all genes (see Choosing an appropriate number of knots in the tradeSeq paper [1]). We follow their vignette and obtain K = 4 in Fig. 3a and K = 6 in Fig. 3f. In tradeSeq, the null distribution of the test statistic Sj is*

*Therefore, when K = 4 (Fig. 3a), the null distribution of every gene is χ23; when K = 6 (Fig. 3a), the null distribution of every gene is χ25. As a result, the distributions of tradeSeq p-values under the null are quite different between Fig. 3a and Fig. 3f. In contrast, PseudotimeDE and NBAMSeq use their default K values, which are not data adaptive, so their p-value distributions are the same in Fig. 3a and Fig. 3f.*

*We should notice that this K selection in tradeSeq is unnecessary and problematic. For the GAM model, “exact choice of K is not generally critical [2]”, if the GAM model is correctly used for inference. This is why we do not include the K selection step in PseudotimeDE. In contrast, the results of tradeSeq is highly sensitive to the choice of K due to their incorrect null distribution. That is, tradeSeq uses the nominal degree of freedom K − 1 in their null distribution (χ2K−1); however, the correct way is to use* ***effect degree of freedom****, which is* ***gene-specific*** *and always* ***smaller*** *than K − 1 due to the penalization on the likelihood function. As a result, tradeSeq uses null distributions (of its test statistics) that have heavier right tails than those of the correct null distributions, resulting in conservative p-values (whose distribution under the null has a mode near 1). Details about the theory and usage of effect degree of freedom in GAM can be found in Dr. Simon Woods’ book [3] and 2013 Biometrika paper [4].*

*Figure R2: Goodness-of-fit of the parametric distribution. The -values are from Anderson-Darling (AD) test, which measures the goodness-of-fit of the gamma/two-component gamma mix-ture distribution to the empirical null distribution generated by subsampling and permutation. (a) Histogram of AD test -values based on the synthetic high dispersion dataset and pseudotime inferred by Slingshot. The distribution is approximately Uniform[0 1], indicating that the para-metric distribution fits the empirical null distribution well. (b) Quantile-quantile plots comparing the empirical null distribution and its corresponding parametric fit for four random genes. (c) Histogram of AD test -values based on the LPS-dendritic cell dataset and pseudotime inferred by Slingshot. The distribution is approximately Uniform[0 1], indicating that the parametric distri-bution fits the empirical null distribution well. (d) Quantile-quantile plots comparing the empirical null distribution and its corresponding parametric fit for four random genes.*

Comment R1.4 As the authors mentioned in the introduction, some other methods (e.g. trade-Seq) also implemented NB-GAM for DE inference. For model fitting of NB-GAM, what is the difference between PseudotimeDE and other mentioned methods? It would be great if the authors can provide some discussion.

*Answer to R1.4*

*PseudotimeDE, tradeSeq and NBAMSeq all rely on R package mgcv for fitting NB-GAM by penalized-restricted maximum likelihood. Therefore, the model fitting of NB-GAM is overall similar in the three methods. There are only minor differences, listed below.*

*1.	In PseudotimeDE, the dispersion parameter of gene is estimated simultaneously with the mean parameter i of gene in cell by the penalized-restricted maximum likelihood (PRML); in contrast, NBAMSeq uses the maximum a posterior (MAP) dispersion estimate by R package DESeq2 [5]. NBAMSeq is designed for bulk RNA-seq and accounts for a samll sample size (i.e., number of replicates); therefore, the MAP estimate by DESeq2 is more biased (for variance reduction purpose) than the PRML estimate used by PseudotimeDE. Given that scRNA-seq has a large enough sample size (i.e., number of cells), we think that the PRML estimate of is preferred over the MAP estimate.*

*2.	In PseudotimeDE, the number of knots is pre-defined as a fix number (the default = 6, which is usually large enough); In contrast, tradeSeq randomly subsamples a small set of genes to choose a for all genes. We have discussed the drawbacks of this selection in Answer to R1.3.*

*3.	PseudotimeDE fits a NB-GAM to each lineage, while tradeSeq incorporates a lineage covari-ate so that it can fit a NB-GAM to multiple lineages jointly. The reason is that PseudotimeDE needs to account for the uncertainty in pseudotime inference and within-lineage model fitting makes the inference easier.*

*4.	Pseudotime also implements a zero-inflated NB-GAM (ZINB-GAM) that includes a dropout probability parameter for each gene, and PseudotimeDE estimates this parameter internally. In contrast, while tradeSeq can also turn its NB-GAM into ZINB-GAM, it estimates the dropout probability parameters externally by ZINB-WaVE [6]; NBAMSeq is designed for bulk RNA-seq data and does not consider zero inflation.*

*Although the NB-GAM fitting of PseudotimeDE is similar to that of the other two methods, its inference (-value calculation) is totally different, and this is the main novelty of PseudotimeDE.*

Comment R1.5 On the Github page for the software, only the Rmd vignette file is provided. It would be good to provide Html/pdf vignettes.

*Answer to R1.5 We thank the reviewer for this nice suggestion. We have posted an HTML ver¬sion of the vignette under the same Github page https://github.com/SONGDONGYUAN1994/ PseudotimeDE.*

Comment R1.6 Typo in figure 3: “FDP” → “FDR”

*Answer to R1.6 Here the FDP is not a typo. It stands for false discovery proportion, defined as the proportion of false discoveries among the discoveries in one synthetic dataset. FDR is the expectation of FDP. Since some other DE methods fail to control their FDR to the nominal 5% level, we set their FDP to 5% to make a fair comparison of their power.*

Answers to Reviewer 2

The approach is well designed, catering to an important question in single-cell analysis and method would be potentially useful to the community.

*We thank the reviewer for appreciating the merit of our work.*

There are a few concerns stated below:

Comment R2.1 It is critical that the authors demonstrate their approach on additional real/published datasets with multiple trajectories and/or lineages.

*Answer to R2.1 We thank the reviewer for this important suggestion. We add two additional real datasets. The first dataset contains three cell lineages (Fig. R3), and the second one contains a cyclic cell lineage (i.e., the cell cycle) (Fig. R4). We acknowledge that PseudotimeDE may not work well for datasets with a complex lineage structure (e.g., a large number of lineages), and we have discussed this issue in the Discussion section as follows.*

*“Due to this topology uncertainty issue, PseudotimeDE is most suitable for single-cell gene expression data that contain only one cell lineage (including cyclic data) or a small number of well separated cell lineages (e.g., bifurcation). The reason is that these data can maintain stable inferred cell topology after subsampling, an essential requirement of PseudotimeDE. That said, PseudotimeDE is not designed for data with many equivocal cell lineages or a complex cell hier¬archy, the data that cannot maintain stable inferred cell topology across subsamples, because for such data, it is difficult to find one-to-one matches between cell lineages inferred from a subsample and those inferred from the original data.”*

*PseudotimeDE outperforms tradeSeq and Monocle3-DE on both new datasets. Here are our new results added to the revised manuscript:*

*Real data example 4: natural killer T cell subtypes*

*“In the fourth application, we compare PseudotimeDE with tradeSeq and Monocle3-DE on a dataset of natural killer T cell (NKT cell) subtypes [7]. We apply Slingshot with PCA for di¬mensionality reduction to infer cell pseudotime and construct the trifurcation topology (Fig. R3a) reported in the original study. We apply the three DE methods to identify DE genes in each of the three lineages. Consistent with the previous results, the -values of tradeSeq follow a bimodal distribution, suggesting that many of them are incorrectly inflated (Fig. R3b).”*

*“For validation purpose, we utilize the lineage-specific DE genes identified from bulk RNA-seq data in the original study [7] to evaluate the DE gene rankings established by PseudotimeDE, tradeSeq, and Monocle3-DE from scRNA-seq data. Specifically, we perform the GSEA analysis using the bulk DE gene sets in the same way as for the pancreatic beta cell maturation dataset. The GSEA analysis shows that PseudotimeDE’s -values best agree with the lineage-specific DE genes from bulk data and thus most distinguish the three lineages. For example, for the NKT1 lineage, PseudotimeDE’s small -values are exclusively enriched in the “NKT1 bulk” gene set, while tradeSeq and Monocle3-DE have small -values enriched in at least two lineage-specific DE gene sets (Fig. R3c). This result confirms that, compared with the DE genes identified by the other two DE methods, the top DE genes identified by PseudotimeDE are more biologically meaningful.”*

*Real data example 5: cell cycle phases*

*“In the fifth application, we compare PseudotimeDE with tradeSeq and Monocle3-DE on a dataset of human induced pluripotent stem cells (iPSCs) measured with cell cycle phases (FUCCI labels) [8]. The original study has reported 101 cyclic genes whose expression levels have large proportions of variance explained (PVE) explained by cells’ FUCCI labels [8]; that is, cells’ FUCCI labels are regarded as the predictor, a gene’s expression levels in the same cells are regarded as the response, and a PVE is calculated from a nonparametric smoothing fit; hence, the larger the PVE, the better the gene’s expression levels can be predicted by the cell cycle phases. The original study has also developed an R package peco to infer cell cycle phases from scRNA-seq data.”*

*“In our study, we first construct a benchmark dataset by treating the 101 cyclic genes as true DE genes and using the same genes with expression levels randomly shuffled across cells as the true non-DE genes; hence, our positive and negative sets both contain 101 genes. Then we apply the R package peco to this dataset to infer each cell’s cycle phase, which is equivalent to pseudotime; that is, we use peco as the pseudotime inference method. Finally, we apply the three DE methods.”*

*“Our results show that, for the true non-DE genes, only PseudotimeDE generates valid -values that approximately follow the Uniform[0 1] distribution (Fig. R4a & c). For the true DE genes, PseudotimeDE’s (− log10 transformed) -values, one per gene, have the highest correlation with these genes’ PVE, indicating that PseudotimeDE successfully identifies the top DE genes as those with the strongest cyclic trends (Fig. R4b). PseudotimeDE also yields successful FDR control, the highest AUROC value, and the highest power, among the three DE methods (Fig. R4d, e & f). Therefore, we conclude that PseudotimeDE outperforms tradeSeq and Monocle3-DE in identifying cell cycle-related genes from this iPSC scRNA-seq dataset.”*

Comment R2.2 While the authors leave the choice of topology/lineage to users, it would be beneficial to further demonstrate suitability of PseudotimeDE to cyclic, linear, converging/binary real datasets

*Figure R3: Application of PseudotimeDE, tradeSeq, and Monocle3-DE to the natural killer T cell dataset. (a) PCA visualization and inferred pseudotime by Slingshot. Pre-defined NKT subtypes are marked by colors. Slingshot returns a trifurcation topology, where the three lineages are NKT0 to NKT1, NKT0 to NKT17, and NKT0 to NKT2. (b) Histograms of all genes’ -values in the three lineages calculated by the three DE methods. (c) Heatmaps of normalized enrichment scores (NESs, marked by colors) and their corresponding -values (in numbers) from the GSEA analysis. Each NES value and its corresponding -value are calculated for each DE method and each lineage, based on the -values of a DE method for a lineage and that lineage’s DE genes found from bulk RNA-seq data, denoted by “NKT1 bulk”, “NKT17 bulk,” or “NKT2 bulk” [7]. Note that among the three DE methods, PseudotimeDE outputs -values that best agree with the lineage-specific DE genes from bulk data and thus most distinguish the three lineages. For instance, for the NKT1 lineage, PseudotimeDE’s small -values are enriched in the “NKT1 bulk” gene set only, while tradeSeq and Monocle3-DE have small -values enriched in at least two lineage-specific DE gene sets.*

*Figure R4: Application of PseudotimeDE, tradeSeq, and Monocle3-DE to the cell cycle phase dataset. (a) Distributions of non-DE genes’ -values by three DE methods with inferred pseudotime. Top: quantile-quantile plots that compare the empirical quantiles of non-DE genes’ -values against the expected quantiles of the Uniform[0 1] distribution. Bottom: histograms of non-DE genes’ -values. The -values shown on top of histograms are from the Kolmogorov–Smirnov test under the null hypothesis that the distribution is Uniform[0 1]. The larger the -value, the more uniform the distribution is. Among the three DE methods, PseudotimeDE’s -values follow most closely the expected Uniform[0 1] distribution. (b) Distributions of DE genes’ -values by three DE methods with inferred pseudotime. Top: scatter plots of DE genes’ -values against the proportions of variance explained (PVE), which measure the strengths of genes’ inferred cyclic trends in the original study [8]. PseudotimeDE’s -values (− log10 transformed) have the highest correlation with the PVE, indicating that PseudotimeDE identifies the genes with the strongest cyclic trends as the top DE genes. Bottom: histograms of all genes’ -values. Blue and red colors represent the -values of DE genes and non-DE genes (same as in (a) bottom), respectively. PseudotimeDE yields the best separation of the two gene groups’ -values. (c) Quantile-quantile plots of the same -values as in (a) on the negative log10 scale. PseudotimeDE returns the best-calibrated -values. (d) FDPs of the three DE methods with the target FDR 005 (BH adjusted-≤005). (e) ROC curves and AUROC values of the three DE methods. PseudotimeDE achieves the highest AUROC. (f) Power of the three DE methods under the FDP = 0.05 cutoff. PseudotimeDE achieves the highest power.*

*Answer to R2.2 We thank the reviewer for this insight comment. As we said in Answer to R2.1, we have added the following text to the Discussion section about PseudotimeDE’s applicability.*

*“Due to this topology uncertainty issue, PseudotimeDE is most suitable for single-cell gene expression data that contain only one cell lineage (including cyclic data) or a small number of well separated cell lineages (e.g., bifurcation). The reason is that these data can maintain stable inferred cell topology after subsampling, an essential requirement of PseudotimeDE. That said, PseudotimeDE is not designed for data with many equivocal cell lineages or a complex cell hier¬archy, the data that cannot maintain stable inferred cell topology across subsamples, because for such data, it is difficult to find one-to-one matches between cell lineages inferred from a subsample and those inferred from the original data. Then, a practical solution for such data is to first define a cell lineage of interest and then apply PseudotimeDE to only the cells assigned to this lineage.”*

Comment R2.3 The authors should also show a few examples of new/predicted DE genes from analysed datasets in a tSNE/UMAP to visually assess heterogeneity between dropouts and ex¬pressed single-cells.

*Answer to R2.3 Thanks to this comment, we have added four figures to visualize a few known DE genes (reported by the original studies as examples) and several new DE genes (identified by PseudotimeDE but missed by tradeSeq or Monocle3-DE) in multiple real datasets. Here we show one figure as an example (Fig. R5). Below is our newly added text.*

*“For illustration purpose, we visualize the expression levels of some known and novel DE genes identified by PseudotimeDE using UMAP, and clear DE patterns are observed.”*

Comment R2.4 In Fig 4, its unclear how there are more exclusive GO terms captured by Pseu-dotimeDE (195 unique DE genes) in comparison to Monocle3 (172 unique DE genes)

*Answer to R2.4 We thank the reviewer’s careful inspection. We have two reasons for the higher number of GO terms by PseudotimeDE’s DE genes. First, the numbers of DE genes found by PseudotimeDE vs. Monocle3-DE are not 195 vs. 172, but 358 vs. 174.*

*“Hence, our second strategy is to probe the functions of the DE genes that are uniquely iden¬tified by one method in pairwise comparisons of PseudotimeDE vs. tradeSeq and PseudotimeDE vs. Monocle3-DE.”*

*The DE genes are unique to PseudotimeDE or Monocle3 in the pairwise comparison, and the PseudotimeDE-unique genes are about twice in size of the Monocle3-unique DE genes.*

*Second, we argue that the number of DE genes is not necessarily related to the number of GO terms. It is possible that a smaller gene set is more biologically meaningful than a larger gene set, thus leading to more significant GO terms.*

*Figure R5: UMAP visualization of example DE genes identified by PseudotimeDE, using Slingshot as the pseudotime inference method, in the LPS-dendritic cell dataset. -values returned by PseudotimeDE are reported for all the 16 example genes. (a) Examples of known DE genes, which have been reported as highly confident DE genes in the original study [9]. (b) Examples of new DE genes, which are identified by PseudotimeDE but found as non-DE by either tradeSeq or Monocle3-DE.*

Comment R2.5 Typos (monolce3, Nap15)

*Answer to R2.5 We thank the reviewer for finding the typo “monolce” in our figures. We have fixed the typo of “Monocle3”. The gene Nap1l5 in Figs. S17 & S18 is not a typo; here “l” is the lower letter l. Please check this gene on UniProt:* [*https://www.uniprot.org/uniprot/Q9JJF0*](https://www.uniprot.org/uniprot/Q9JJF0)*.*

*References*

*[1]	Koen Van den Berge, Hector Roux De Bezieux, Kelly Street, Wouter Saelens, Robrecht Can-noodt, Yvan Saeys, Sandrine Dudoit, and Lieven Clement. Trajectory-based differential ex¬pression analysis for single-cell sequencing data. Nature communications, 11(1):1–13, 2020.*

*[2]	Simon N. Wood. Basis dimension choice for smooths. URL https://stat.ethz.ch/R-manual/ R-patched/library/mgcv/html/choose.k.html.*

*[3]	Simon N Wood. Generalized additive models: an introduction with R. CRC press, 2017.*

*[4]	Simon N Wood. On p-values for smooth components of an extended generalized additive model. Biometrika, 100(1):221–228, 2013.*

*[5]	Michael I Love, Wolfgang Huber, and Simon Anders. Moderated estimation of fold change and dispersion for rna-seq data with deseq2. Genome biology, 15(12):550, 2014.*

*[6]	Davide Risso, Fanny Perraudeau, Svetlana Gribkova, Sandrine Dudoit, and Jean-Philippe Vert. A general and flexible method for signal extraction from single-cell rna-seq data. Nature com-munications, 9(1):1–17, 2018.*

*[7]	Isaac Engel, Gre´gory Seumois, Lukas Chavez, Daniela Samaniego-Castruita, Brandie White, Ashu Chawla, Dennis Mock, Pandurangan Vijayanand, and Mitchell Kronenberg. Innate-like functions of natural killer t cell subsets result from highly divergent gene programs. Nature immunology, 17(6):728–739, 2016.*

*[8]	Chiaowen Joyce Hsiao, PoYuan Tung, John D Blischak, Jonathan E Burnett, Kenneth A Barr, Kushal K Dey, Matthew Stephens, and Yoav Gilad. Characterizing and inferring quantitative cell cycle phase in single-cell rna-seq data analysis. Genome research, 30(4):611–621, 2020.*

*[9]	Alex K Shalek, Rahul Satija, Joe Shuga, John J Trombetta, Dave Gennert, Diana Lu, Peilin Chen, Rona S Gertner, Jellert T Gaublomme, Nir Yosef, et al. Single-cell rna-seq reveals dynamic paracrine control of cellular variation. Nature, 510(7505):363–369, 2014.*

**Second round of review**

**Reviewer 1**

All my previous comments are sufficiently addressed. Thank you.

**Reviewer 2**

The authors have addressed most of my concerns.
